# Supplementary material for: Quality suitability regionalization analysis of Angelica sinensis in Gansu, China
Source: PLoS One. 2020 Dec 14;15(12):e0243750. doi: 10.1371/journal.pone.0243750 (PMC7735642; doi:10.1371/journal.pone.0243750)
Supplement: S1 Appendix — (PDF) [file pone.0243750.s001.pdf]

Appendix 1 Sampling point informations

No.	Areas			Longitude	Latitude	Height (m)
	City	County	Township			
1	Dingxi	Longxi	Caizi	104° 43′ 93″	34° 90′ 91″	2137
2	Dingxi	Longxi	Caizi	104° 32′ 95″	34° 94′ 06″	2385
3	Dingxi	Longtao	Yaodian	104° 02′ 50″	35° 16′ 31″	2253
4	Dingxi	Longtao	Shangying	104° 02′ 20″	35° 37′ 36″	2631
5	Dingxi	Longtao	Shangying	104° 02′ 29″	35° 37′ 28″	2676
6	Dingxi	Longtao	Shangying	104° 01′ 48″	35° 37′ 30″	2559
7	Dingxi	Longtao	Shangying	104° 01′ 45″	35° 39′ 53″	2784
8	Dingxi	Longtao	Shangying	104° 01′ 15″	35° 39′ 17″	2802
9	Dingxi	Longtao	Shangying	104° 00′ 31″	35° 39′ 32″	2762
10	Dingxi	Longtao	Shangying	104° 00′ 31″	35° 39′ 52″	2762
11	Dingxi	Longtao	Shangying	104° 00′ 00″	35° 39′ 54″	2699
12	Dingxi	Longtao	Shangying	103° 59′ 13″	35° 39′ 21″	2578
13	Dingxi	Longtao	Shangying	103° 58′ 02″	35° 38′ 31″	2431
14	Dingxi	Longtao	Shangying	103° 59′ 13″	35° 37′ 06″	2434
15	Dingxi	Longtao	Xindian	103° 56′ 46″	35° 40′ 00″	2587
16	Dingxi	Longtao	Nanping	103° 46′ 40″	35° 08′ 44″	2241
17	Dingxi	Longtao	Kangjiaji	103° 59′ 58″	35° 15′ 46″	2322
18	Dingxi	Longtao	Kangjiaji	103° 59′ 21″	35° 16′ 42″	2337
19	Dingxi	Longtao	Kangjiaji	103° 56′ 42″	35° 15′ 23″	2319
20	Dingxi	Longtao	Kangjiaji	103° 56′ 44″	35° 15′ 41″	2331
21	Dingxi	Longtao	Kangjiaji	103° 57′ 13″	35° 15′ 28″	2371
22	Dingxi	Longtao	Kangjiaji	103° 57′ 27″	35° 15′ 16″	2415
23	Dingxi	Longtao	Kangjiaji	103° 58′ 20″	35° 14′ 49″	2415
24	Dingxi	Longtao	Kangjiaji	103° 58′ 52″	35° 15′ 12″	2396
25	Dingxi	Longtao	Kangjiaji	103° 58′ 49″	35° 14′ 02″	2313
26	Dingxi	Longtao	Kangjiaji	103° 58′ 34″	35° 13′ 43″	2335
27	Dingxi	Longtao	Taishi	103° 51′ 57″	35° 46′ 22″	2771
28	Dingxi	Longtao	Taishi	103° 51′ 08″	35° 44′ 17″	2575
29	Dingxi	Longtao	Taishi	103° 52′ 28″	35° 43′ 30″	2662
30	Dingxi	Longtao	Shangying	104° 00′ 24〃	35° 60′ 71〃	2415
31	Dingxi	Longtao	Shangying	104° 00′ 36〃	35° 60′ 65〃	2443
32	Dingxi	Longtao	Shangying	104° 04′ 20〃	35° 62′ 58〃	2670
33	Dingxi	Longtao	Shangying	104° 04′ 28〃	35° 62′ 59〃	2682
34	Dingxi	Longtao	Shangying	104° 03′ 65〃	35° 59′ 39〃	2453
35	Dingxi	Longtao	Shangying	104° 03′ 23〃	35° 58′ 80〃	2468
36	Dingxi	Longtao	Shangying	104° 04′ 84〃	35° 60′ 54〃	2542
37	Dingxi	Longtao	Shangying	104° 04′ 51〃	35° 62′ 27〃	2605
38	Dingxi	Longtao	Shangying	104° 02′ 00〃	35° 59′ 79〃	2473
39	Dingxi	Longtao	Shangying	104° 05′ 78〃	35° 62′ 69〃	2676
40	Dingxi	Longtao	Shangying	104° 05′ 05〃	35° 62′ 62〃	2670
41	Dingxi	Longtao	Shangying	104° 05′ 03〃	35° 62′ 74〃	2686
42	Dingxi	Longtao	Zhongpu	103° 39′ 55〃	35° 54′ 37〃	2696
43	Dingxi	Longtao	Zhongpu	103° 40′ 10〃	35° 55′ 00〃	2644
44	Dingxi	Longtao	Zhongpu	103° 51′ 29〃	35° 55′ 00〃	2703
45	Dingxi	Longtao	Zhongpu	103° 69′ 02〃	35° 97′ 67〃	2671

46	Dingxi	Longtao	Zhongpu	103° 42′ 16〃	35° 54′ 32〃	2672
47	Dingxi	Longtao	Zhongpu	103° 70′ 47〃	35° 90′ 78〃	2645
48	Dingxi	Longtao	Zhongpu	103° 71′ 74〃	35° 89′ 36〃	2539
49	Dingxi	Longtao	Zhongpu	103° 71′ 98〃	35° 96′ 10〃	2623
50	Dingxi	Longtao	Zhongpu	103° 73′ 88〃	35° 87′ 17〃	2562
51	Dingxi	Longtao	Zhongpu	103° 75′ 40〃	35° 89′ 30〃	2559
52	Dingxi	Longtao	Zhongpu	103° 76′ 92〃	35° 89′ 37〃	2435
53	Dingxi	Longtao	Kangjiaji	104° 02′ 11〃	35° 14′ 56〃	2343
54	Dingxi	Longtao	Kangjiaji	104° 02′ 11〃	35° 14′ 29〃	2326
55	Dingxi	Longtao	Kangjiaji	104° 02′ 19〃	35° 13′ 43〃	2412
56	Dingxi	Longtao	Kangjiaji	104° 02′ 34〃	35° 13′ 37〃	2385
57	Dingxi	Longtao	Kangjiaji	104° 03′ 46〃	35° 13′ 54〃	2349
58	Dingxi	Longtao	Kangjiaji	104° 01′ 51〃	35° 13′ 29〃	2415
59	Dingxi	Longtao	Kangjiaji	104° 01′ 16〃	35° 12′ 38〃	2407
60	Dingxi	Longtao	Kangjiaji	104° 01′ 13〃	35° 12′ 32〃	2436
61	Dingxi	Longtao	Kangjiaji	104° 01′ 07〃	35° 12′ 32〃	2419
62	Dingxi	Longtao	Kangjiaji	104° 01′ 03〃	35° 12′ 34〃	2411
63	Dingxi	Longtao	Kangjiaji	103° 59′ 54〃	35° 13′ 58〃	2181
64	Dingxi	Longtao	Kangjiaji	104° 00′ 10〃	35° 12′ 47〃	2223
65	Dingxi	Longtao	Kangjiaji	104° 00′ 08〃	35° 12′ 47〃	2228
66	Dingxi	Longtao	Longmen	103° 56′ 56〃	35° 16′ 19〃	2236
67	Dingxi	Longtao	Xiakou	104° 10′ 54〃	35° 62′ 07〃	2579
68	Dingxi	Longtao	Xiakou	104° 09′ 14〃	35° 62′ 19〃	2588
69	Dingxi	Longtao	Xiakou	104° 09′ 12〃	35° 61′ 68〃	2497
70	Dingxi	Longtao	Xiakou	103° 98′ 96〃	35° 53′ 66〃	2055
71	Dingxi	Weiyuan	Qingyuan	104° 26′	35° 14′	2053
72	Dingxi	Weiyuan	Qingyuan	104° 26′	35° 14′	2056
73	Dingxi	Weiyuan	Qingyuan	104° 17′ 41″	35° 10′ 35″	2313
74	Dingxi	Weiyuan	Huichuan	103° 57′ 50″	35° 08′ 20″	2332
75	Dingxi	Weiyuan	Huichuan	103° 58′ 48″	35° 08′ 59″	2331
76	Dingxi	Weiyuan	Huichuan	103° 59′ 09″	35° 07′ 47″	2174
77	Dingxi	Weiyuan	Huichuan	104° 00′ 00″	35° 05′ 53″	2294
78	Dingxi	Weiyuan	Huichuan	103° 59′ 16″	35° 06′ 26″	2211
79	Dingxi	Weiyuan	Huichuan	103° 57′ 59″	35° 06′ 19″	2270
80	Dingxi	Weiyuan	Huichuan	103° 58′ 38″	35° 05′ 19″	2266
81	Dingxi	Weiyuan	Huichuan	103° 58′ 26″	35° 04′ 50″	2287
82	Dingxi	Weiyuan	Huichuan	103° 58′ 46″	35° 02′ 53″	2363
83	Dingxi	Weiyuan	Huichuan	103° 58′ 46″	35° 02′ 51″	2367
84	Dingxi	Weiyuan	Huichuan	103° 58′ 49″	35° 02′ 23″	2371
85	Dingxi	Weiyuan	Huichuan	103° 58′ 54″	35° 00′ 41″	2428
86	Dingxi	Weiyuan	Huichuan	103° 58′ 46″	35° 01′ 42″	2442
87	Dingxi	Weiyuan	Qijiamiao	104° 03′ 00″	35° 07′ 26″	2359
88	Dingxi	Weiyuan	Qijiamiao	104° 02′ 16″	35° 07′ 32″	2480
89	Dingxi	Weiyuan	Qijiamiao	104° 03′ 03″	35° 09′ 34″	2465
90	Dingxi	Weiyuan	Qijiamiao	104° 01′ 46″	35° 10′ 46″	2513
91	Dingxi	Weiyuan	Qijiamiao	104° 00′ 19″	35° 09′ 05″	2295
92	Dingxi	Weiyuan	Qiuyu	104° 11′ 10″	35° 02′ 29″	2227
93	Dingxi	Weiyuan	Qiuyu	104° 11′ 01″	35° 04′ 48″	2228

94	Dingxi	Weiyuan	Qiuyu	104° 08′ 44″	35° 59′ 44″	2485
95	Dingxi	Weiyuan	Qiuyu	104° 08′ 45″	35° 59′ 45″	2600
96	Dingxi	Weiyuan	Shangwan	103° 55′ 21″	35° 09′ 51″	2279
97	Dingxi	Weiyuan	Shangwan	103° 55′ 36″	35° 09′ 08″	2297
98	Dingxi	Weiyuan	Shangwan	103° 55′ 07″	35° 06′ 58″	2133
99	Dingxi	Weiyuan	Shangwan	103° 54′ 40″	35° 08′ 44″	2190
100	Dingxi	Weiyuan	Tianjiahe	103° 58′ 24″	35° 02′ 24″	2411
101	Dingxi	Weiyuan	Tianjiahe	103° 58′ 19″	35° 02′ 15″	2426
102	Dingxi	Weiyuan	Tianjiahe	103° 58′ 17″	35° 01′ 58″	2445
103	Dingxi	Weiyuan	Tianjiahe	103° 53′ 55″	35° 03′ 34″	2297
104	Dingxi	Weiyuan	Tianjiahe	103° 53′ 56″	35° 03′ 35″	2322
105	Dingxi	Weiyuan	Tianjiahe	103° 55′ 12″	35° 06′ 28″	2156
106	Dingxi	Weiyuan	Tianjiahe	103° 56′ 41″	35° 01′ 51″	2490
107	Dingxi	Weiyuan	Tianjiahe	103° 56′ 37″	35° 01′ 54″	2504
108	Dingxi	Weiyuan	Wuzhu	104° 14′ 06″	35° 18′ 55″	2195
109	Dingxi	Weiyuan	Wuzhu	104° 06′ 38″	35° 04′ 30″	2265
110	Dingxi	Weiyuan	Wuzhu	104° 05′ 55″	35° 04′ 43″	2310
111	Dingxi	Weiyuan	Wuzhu	104° 05′ 28″	35° 05′ 05″	2264
112	Dingxi	Weiyuan	Wuzhu	104° 05′ 23″	35° 05′ 17″	2254
113	Dingxi	Weiyuan	Wuzhu	104° 05′ 05″	35° 03′ 55″	2318
114	Dingxi	Weiyuan	Wuzhu	104° 05′ 06″	35° 03′ 56″	2367
115	Dingxi	Weiyuan	Wuzhu	104° 04′ 40″	35° 03′ 03″	2342
116	Dingxi	Weiyuan	Wuzhu	104° 04′ 59″	35° 02′ 57″	2420
117	Dingxi	Weiyuan	Wuzhu	104° 04′ 09″	35° 01′ 51″	2353
118	Dingxi	Weiyuan	Wuzhu	104° 04′ 13″	35° 01′ 30″	2359
119	Dingxi	Weiyuan	Wuzhu	104° 04′ 07″	35° 01′ 06″	2375
120	Dingxi	Weiyuan	Wuzhu	104° 04′ 08″	35° 01′ 53″	2398
121	Dingxi	Weiyuan	Wuzhu	104° 04′ 38″	35° 02′ 01″	2335
122	Dingxi	Weiyuan	Wuzhu	104° 05′ 35″	35° 01′ 22″	2330
123	Dingxi	Weiyuan	Wuzhu	104° 06′ 09″	35° 01′ 49″	2277
124	Dingxi	Weiyuan	Wuzhu	104° 06′ 04″	35° 01′ 30″	2294
125	Dingxi	Weiyuan	Wuzhu	104° 07′ 52″	35° 00′ 38″	2396
126	Dingxi	Weiyuan	Wuzhu	104° 07′ 39″	35° 00′ 40″	2382
127	Dingxi	Weiyuan	Wuzhu	104° 08′	47° 91′	2221
128	Dingxi	Weiyuan	Qingyuan	104° 11′ 94″	35° 11′ 82″	2407
129	Dingxi	Weiyuan	Qingyuan	104° 15′ 09″	35° 15′ 32″	2383
130	Dingxi	Weiyuan	Qingyuan	104° 20′ 94″	35° 15′ 47″	2132
131	Dingxi	Weiyuan	Qingyuan	104° 18′ 56″	35° 17′ 52″	2286
132	Dingxi	Weiyuan	Qingyuan	104° 21′ 72″	35° 17′ 82″	2357
133	Dingxi	Weiyuan	Qingyuan	104° 19′ 62″	35° 19′ 06″	2372
134	Dingxi	Weiyuan	Qingyuan	104° 15′ 92″	35° 18′ 51″	2357
135	Dingxi	Weiyuan	Qingyuan	104° 12′ 83″	35° 10′ 43″	2220
136	Dingxi	Weiyuan	Qingyuan	104° 10′ 84″	35° 08′ 02″	2359
137	Dingxi	Weiyuan	Pengfeng	104° 22′ 47″	35° 97′ 87″	2295
138	Dingxi	Weiyuan	Pengfeng	104° 24′ 40″	35° 96′ 76″	2339
139	Dingxi	Weiyuan	Pengfeng	104° 25′ 99″	35° 96′ 14″	2392
140	Dingxi	Weiyuan	Huichuan	104° 98′ 45″	35° 27′ 04″	2332
141	Dingxi	Weiyuan	Huichuan	104° 00′ 25″	35° 11′ 15″	2244

142	Dingxi	Weiyuan	Huichuan	104° 01′ 19″	35° 11′ 74″	2365
143	Dingxi	Weiyuan	Huichuan	104° 02′ 12″	35° 09′ 98″	2284
144	Dingxi	Weiyuan	Huichuan	104° 02′ 64″	35° 09′ 71″	2296
145	Dingxi	Weiyuan	Huichuan	104° 03′ 66″	35° 07′ 80″	2336
146	Dingxi	Weiyuan	Huichuan	104° 03′ 74″	35° 07′ 65″	2344
147	Dingxi	Weiyuan	Huichuan	104° 04′ 54″	35° 06′ 09″	2380
148	Dingxi	Weiyuan	Huichuan	104° 05′ 19″	35° 05′ 26″	2413
149	Dingxi	Weiyuan	Huichuan	104° 05′ 20″	35° 05′ 20″	2413
150	Dingxi	Weiyuan	Huichuan	104° 05′ 20″	35° 05′ 20″	2412
151	Dingxi	Weiyuan	Huichuan	104° 03′ 20″	35° 02′ 55″	2570
152	Dingxi	Weiyuan	Huichuan	104° 02′ 88″	35° 05′ 60″	2442
153	Dingxi	Weiyuan	Huichuan	104° 01′ 97″	35° 05′ 04″	2387
154	Dingxi	Weiyuan	Huichuan	104° 01′ 91″	35° 05′ 08″	2395
155	Dingxi	Weiyuan	Huichuan	104° 01′ 37″	35° 05′ 95″	2451
156	Dingxi	Weiyuan	Huichuan	104° 01′ 12″	35° 05′ 92″	2468
157	Dingxi	Weiyuan	Huichuan	104° 00′ 19″	35° 05′ 59″	2403
158	Dingxi	Weiyuan	Huichuan	104° 99′ 79″	35° 06′ 27″	2421
159	Dingxi	Weiyuan	Beizhai	104° 27′ 60″	35° 23′ 16″	2294
160	Dingxi	Weiyuan	Beizhai	104° 29′ 66″	35° 21′ 10″	2269
161	Dingxi	Weiyuan	Qingping	104° 10′ 57″	35° 18′ 95″	2165
162	Dingxi	Weiyuan	Qingping	104° 11′ 31″	35° 15′ 09″	2356
163	Dingxi	Weiyuan	Qingping	104° 13′ 70″	35° 15′ 58″	2388
164	Dingxi	Weiyuan	Qingping	104° 12′ 72″	35° 16′ 52″	2297
165	Dingxi	Weiyuan	Qingping	104° 08′ 05″	35° 20′ 13″	2392
166	Dingxi	Weiyuan	Qingping	104° 07′ 45″	35° 19′ 69″	2422
167	Dingxi	Weiyuan	Qingping	104° 06′ 54″	35° 18′ 40″	2482
168	Dingxi	Weiyuan	Qijiamiao	104° 07′ 71″	35° 10′ 66″	2374
169	Dingxi	Weiyuan	Qijiamiao	104° 06′ 79″	35° 09′ 80″	2424
170	Dingxi	Weiyuan	Qijiamiao	104° 09′ 00″	35° 10′ 91″	2293
171	Dingxi	Weiyuan	Qijiamiao	104° 10′ 04″	35° 13′ 01″	2257
172	Dingxi	Weiyuan	Qijiamiao	104° 06′ 06″	35° 10′ 50″	2358
173	Dingxi	Weiyuan	Qijiamiao	104° 06′ 07″	35° 10′ 00″	2357
174	Dingxi	Weiyuan	Qijiamiao	104° 07′ 12″	35° 10′ 09″	2436
175	Dingxi	Weiyuan	Qijiamiao	104° 07′ 27″	35° 10′ 17″	2434
176	Dingxi	Weiyuan	Qijiamiao	104° 07′ 81″	35° 10′ 81″	2391
177	Dingxi	Weiyuan	Qijiamiao	104° 10′ 00″	35° 13′ 16″	2256
178	Dingxi	Weiyuan	Qijiamiao	104° 06′ 88″	35° 14′ 46″	2266
179	Dingxi	Weiyuan	Qijiamiao	104° 06′ 58″	35° 14′ 56″	2260
180	Dingxi	Weiyuan	Qijiamiao	104° 05′ 05″	35° 13′ 79″	2322
181	Dingxi	Weiyuan	Qijiamiao	104° 06′ 12″	35° 14′ 64″	2268
182	Dingxi	Weiyuan	Qijiamiao	104° 07′ 19″	35° 14′ 09″	2244
183	Dingxi	Weiyuan	Shangwan	103° 99′ 19″	35° 16′ 85″	2250
184	Dingxi	Weiyuan	Shangwan	103° 00′ 38″	35° 17′ 06″	2299
185	Dingxi	Weiyuan	Shangwan	103° 00′ 40″	35° 17′ 35″	2353
186	Dingxi	Weiyuan	Shangwan	103° 95′ 77″	35° 17′ 42″	2154
187	Dingxi	Weiyuan	Shangwan	103° 95′ 42″	35° 18′ 09″	2132
188	Dingxi	Weiyuan	Shangwan	103° 94′ 15″	35° 22′ 76″	2302
189	Dingxi	Weiyuan	Shangwan	103° 93′ 61″	35° 24′ 02″	2218

190	Dingxi	Weiyuan	Shangwan	103° 89′ 55″	35° 21′ 10″	2015
191	Dingxi	Weiyuan	Shangwan	103° 94′ 82″	35° 19′ 94″	2094
192	Dingxi	Weiyuan	Shangwan	103° 92′ 49″	35° 21′ 68″	2050
193	Dingxi	Weiyuan	Shangwan	103° 93′ 96″	35° 22′ 78″	2316
194	Dingxi	Weiyuan	Majiaji	103° 88′ 04〃	35° 11′ 06〃	2349
195	Dingxi	Weiyuan	Majiaji	103° 87′ 75〃	35° 11′ 46〃	2401
196	Dingxi	Weiyuan	Majiaji	103° 86′ 96〃	35° 09′ 90〃	2224
197	Dingxi	Weiyuan	Majiaji	103° 80′ 77〃	35° 10′ 47〃	2207
198	Dingxi	Weiyuan	Shangwan	103° 89′ 69〃	35° 21′ 10〃	2021
199	Dingxi	Zhangxian	Yihuqiao	104° 24′ 82″	34° 83′ 52″	2279
200	Dingxi	Zhangxian	Yihuqiao	104° 25′ 07″	34° 83′ 48″	2263
201	Dingxi	Zhangxian	Yihuqiao	104° 26′ 68″	34° 83′ 12″	2155
202	Dingxi	Zhangxian	Dongquan	104° 61′ 57″	34° 57′ 51″	2199
203	Dingxi	Zhangxian	Dongquan	104° 59′ 53″	34° 56′ 63″	2329
204	Dingxi	Zhangxian	Dongquan	104° 53′ 70″	34° 54′ 30″	2427
205	Dingxi	Zhangxian	Dongquan	104° 53′ 69″	34° 54′ 24″	2437
206	Dingxi	Zhangxian	Dongquan	104° 53′ 74″	34° 54′ 20″	2448
207	Dingxi	Zhangxian	Dongquan	104° 54′ 94″	34° 52′ 36″	2523
208	Dingxi	Zhangxian	Dongquan	104° 56′ 27″	34° 52′ 40″	2581
209	Dingxi	Zhangxian	Dongquan	104° 60′ 87″	34° 54′ 23″	2483
210	Dingxi	Zhangxian	Dongquan	104° 65′ 10″	34° 58′ 52″	2259
211	Dingxi	Zhangxian	Jinzhong	104° 22′ 19″	34° 79′ 97″	2219
212	Dingxi	Zhangxian	Jinzhong	104° 21′ 93″	34° 79′ 96″	2217
213	Dingxi	Zhangxian	Jinzhong	104° 21′ 16″	34° 80′ 00″	2242
214	Dingxi	Zhangxian	Jinzhong	104° 20′ 25″	34° 79′ 90″	2259
215	Dingxi	Zhangxian	Jinzhong	104° 20′ 31″	34° 79′ 90″	2244
216	Dingxi	Zhangxian	Jinzhong	104° 20′ 04″	34° 79′ 91″	2240
217	Dingxi	Zhangxian	Jinzhong	104° 17′ 13″	24° 80′ 21″	2326
218	Dingxi	Zhangxian	Jinzhong	104° 17′ 15″	24° 80′ 19″	2328
219	Dingxi	Zhangxian	Jinzhong	104° 17′ 10″	34° 80′ 17″	2331
220	Dingxi	Zhangxian	Jinzhong	104° 16′ 70″	34° 80′ 76″	2385
221	Dingxi	Zhangxian	Jinzhong	104° 16′ 96″	34° 81′ 43″	2446
222	Dingxi	Zhangxian	Jinzhong	104° 16′ 94″	34° 81′ 41″	2454
223	Dingxi	Zhangxian	Jinzhong	104° 15′ 45″	34° 80′ 50″	2353
224	Dingxi	Zhangxian	Jinzhong	104° 13′ 46″	34° 81′ 48″	2410
225	Dingxi	Zhangxian	Jinzhong	104° 13′ 08″	34° 81′ 66″	2421
226	Dingxi	Zhangxian	Jinzhong	104° 12′ 39″	34° 81′ 78″	2447
227	Dingxi	Zhangxian	Jinzhong	104° 12′ 14″	34° 81′ 86″	2444
228	Dingxi	Zhangxian	Jinzhong	104° 12′ 08″	34° 81′ 77″	2453
229	Dingxi	Zhangxian	Jinzhong	104° 09′ 94″	34° 82′ 25″	2483
230	Dingxi	Zhangxian	Jinzhong	104° 08′ 86″	34° 82′ 39″	2528
231	Dingxi	Zhangxian	Jinzhong	104° 08′ 79″	24° 82′ 37″	2514
232	Dingxi	Zhangxian	Jinzhong	104° 05′ 68″	34° 83′ 22″	2661
233	Dingxi	Zhangxian	Jinzhong	104° 05′ 44″	34° 83′ 29″	2662
234	Dingxi	Zhangxian	Jinzhong	104° 04′ 93″	34° 83′ 52″	2673
235	Dingxi	Zhangxian	Jinzhong	104° 02′ 42″	34° 87′ 74″	2796
236	Dingxi	Zhangxian	Jinzhong	104° 02′ 09″	34° 85′ 02″	2825
237	Dingxi	Zhangxian	Jinzhong	104° 02′ 12″	34° 84′ 99″	2825

238	Dingxi	Zhangxian	Jinzhong	104° 00′ 47″	34° 85′ 14″	2931
239	Dingxi	Zhangxian	Jinzhong	104° 00′ 24″	34° 85′ 08″	2947
240	Dingxi	Zhangxian	Jinzhong	104° 00′ 24″	34° 85′ 03″	2936
241	Dingxi	Zhangxian	Jinzhong	104° 02′ 95″	34° 85′ 67″	2918
242	Dingxi	Zhangxian	Jinzhong	104° 02′ 99″	34° 85′ 85″	2954
243	Dingxi	Zhangxian	Jinzhong	104° 02′ 99″	34° 85′ 89″	2930
244	Dingxi	Zhangxian	Jinzhong	104° 13′ 42″	34° 81′ 12″	2395
245	Dingxi	Zhangxian	Jinzhong	104° 11′ 00″	34° 80′ 16″	2461
246	Dingxi	Zhangxian	Jinzhong	104° 11′ 01″	34° 80′ 14″	2466
247	Dingxi	Zhangxian	Jinzhong	104° 10′ 50″	34° 79′ 94″	2480
248	Dingxi	Zhangxian	Jinzhong	104° 10′ 54″	34° 79′ 97″	2481
249	Dingxi	Zhangxian	Jinzhong	104° 10′ 47″	34° 79′ 88″	2480
250	Dingxi	Zhangxian	Yihuqiao	104° 33′ 52″	34° 79′ 18″	2403
251	Dingxi	Zhangxian	Yihuqiao	104° 36′ 68″	34° 80′ 55″	2321
252	Dingxi	Zhangxian	Yihuqiao	104° 32′ 84″	34° 79′ 77″	2392
253	Dingxi	Zhangxian	Yihuqiao	104° 32′ 07″	34° 79′ 92″	2112
254	Dingxi	Zhangxian	Yihuqiao	104° 34′ 91″	34° 77′ 75″	2313
255	Dingxi	Zhangxian	Yihuqiao	104° 34′ 63″	34° 77′ 77″	2476
256	Dingxi	Zhangxian	Yanjing	104° 35′ 09″	34° 78′ 58″	2528
257	Dingxi	Zhangxian	Yanjing	104° 35′ 94″	34° 78′ 34″	2327
258	Dingxi	Zhangxian	Dacaotan	104° 16′ 64″	34° 67′ 41″	2623
259	Dingxi	Zhangxian	Dacaotan	104° 16′ 88″	34° 67′ 18″	2630
260	Dingxi	Zhangxian	Dacaotan	104° 16′ 87″	34° 67′ 21″	2641
261	Dingxi	Zhangxian	Dacaotan	104° 11′ 22″	34° 68′ 79″	2718
262	Dingxi	Zhangxian	Dacaotan	104° 10′ 80″	34° 69′ 25″	2732
263	Dingxi	Zhangxian	Dacaotan	104° 10′ 69″	34° 69′ 28″	2757
264	Dingxi	Zhangxian	Yihuqiao	104° 26′ 29″	34° 81′ 62″	2116
265	Dingxi	Zhangxian	Yihuqiao	104° 26′ 22″	34° 81′ 20″	2129
266	Dingxi	Zhangxian	Dacaotan	104° 26′ 91″	34° 79′ 58″	2188
267	Dingxi	Zhangxian	Dacaotan	104° 22′ 35″	34° 77′ 25″	2279
268	Dingxi	Zhangxian	Dacaotan	104° 26′ 85″	34° 78′ 72″	2173
269	Dingxi	Zhangxian	Dacaotan	104° 21′ 59″	34° 77′ 10″	2297
270	Dingxi	Zhangxian	Dacaotan	104° 21′ 54″	34° 77′ 09″	2239
271	Dingxi	Zhangxian	Dacaotan	104° 21′ 48″	34° 77′ 14″	2303
272	Dingxi	Zhangxian	Dacaotan	104° 19′ 63″	34° 75′ 43″	2369
273	Dingxi	Zhangxian	Xinsi	104° 59′ 42″	34° 61′ 17″	182
274	Dingxi	Zhangxian	Dacaotan	104° 19′ 61″	34° 75′ 41″	2371
275	Dingxi	Zhangxian	Dacaotan	104° 19′ 44″	34° 75′ 23″	2366
276	Dingxi	Zhangxian	Dacaotan	104° 17′ 95″	34° 74′ 38″	2404
277	Dingxi	Zhangxian	Dacaotan	104° 17′ 40″	34° 74′ 15″	2417
278	Dingxi	Zhangxian	Dacaotan	104° 17′ 39″	34° 74′ 10″	2426
279	Dingxi	Zhangxian	Dacaotan	104° 15′ 61″	34° 68′ 85″	2575
280	Dingxi	Zhangxian	Dacaotan	104° 15′ 38″	34° 69′ 71″	2594
281	Dingxi	Zhangxian	Dacaotan	104° 16′ 43″	34° 72′ 93″	2457
282	Dingxi	Zhangxian	Yanjing	104° 37′ 93″	34° 79′ 23″	2185
283	Dingxi	Zhangxian	Yanjing	104° 38′ 47″	34° 80′ 10″	2112
284	Dingxi	Zhangxian	Yanjing	104° 37′ 40″	34° 80′ 73″	2178
285	Dingxi	Zhangxian	Caotan	104° 55′ 24″	34° 60′ 54″	2340

286	Dingxi	Zhangxian	Caotan	104° 55′ 21″	34° 60′ 57″	2335
287	Dingxi	Zhangxian	Caotan	104° 55′ 29″	34° 60′ 17″	2372
288	Dingxi	Zhangxian	Caotan	104° 52′ 31″	34° 60′ 15″	2375
289	Dingxi	Zhangxian	Caotan	104° 49′ 59″	34° 60′ 52″	2382
290	Dingxi	Zhangxian	Caotan	104° 49′ 59″	34° 60′ 50″	2377
291	Dingxi	Zhangxian	Caotan	104° 48′ 68″	34° 62′ 64″	2356
292	Dingxi	Zhangxian	Caotan	104° 45′ 86″	34° 62′ 85″	2381
293	Dingxi	Zhangxian	Caotan	104° 49′ 48″	34° 58′ 79″	2360
294	Dingxi	Zhangxian	Caotan	104° 49′ 52″	34° 58′ 77″	2356
295	Dingxi	Zhangxian	Caotan	104° 46′ 58″	34° 59′ 45″	2435
296	Dingxi	Zhangxian	Caotan	104° 46′ 65″	34° 59′ 47″	2433
297	Dingxi	Zhangxian	Caotan	104° 44′ 50″	34° 61′ 30″	2355
298	Dingxi	Zhangxian	Caotan	104° 44′ 65″	34° 61′ 27″	2350
299	Dingxi	Zhangxian	Caotan	104° 44′ 73″	34° 61′ 30″	2352
300	Dingxi	Zhangxian	Caotan	104° 44′ 83″	34° 62′ 86″	2366
301	Dingxi	Zhangxian	Caotan	104° 44′ 90″	34° 64′ 22″	2401
302	Dingxi	Zhangxian	Caotan	104° 44′ 92″	34° 64′ 25″	2402
303	Dingxi	Zhangxian	Caotan	104° 44′ 41″	34° 65′ 04″	2398
304	Dingxi	Zhangxian	Sizu	104° 41′ 59″	34° 64′ 85″	2523
305	Dingxi	Zhangxian	Sizu	104° 41′ 53″	34° 64′ 84″	2521
306	Dingxi	Zhangxian	Shichuan	104° 41′ 68″	34° 59′ 04″	2499
307	Dingxi	Zhangxian	Shichuan	104° 41′ 76″	34° 59′ 18″	2514
308	Dingxi	Zhangxian	Shichuan	104° 42′ 30″	34° 60′ 18″	2455
309	Dingxi	Zhangxian	Shichuan	104° 40′ 34″	34° 61′ 08″	2534
310	Dingxi	Zhangxian	Caotan	104° 39′ 80″	34° 61′ 64″	2644
311	Dingxi	Zhangxian	Caotan	104° 39′ 96″	34° 61′ 20″	2574
312	Dingxi	Zhangxian	Shichuan	104° 37′ 54″	34° 59′ 32″	2482
313	Dingxi	Zhangxian	Shichuan	104° 27′ 99″	34° 58′ 65″	2324
314	Dingxi	Zhangxian	Shichuan	104° 32′ 96″	34° 58′ 37″	2221
315	Dingxi	Zhangxian	Shichuan	104° 32′ 98″	34° 58′ 32″	2230
316	Dingxi	Zhangxian	Shichuan	104° 33′ 32″	34° 58′ 31″	2215
317	Dingxi	Zhangxian	Shichuan	104° 32′ 11″	34° 60′ 31″	2162
318	Dingxi	Zhangxian	Shichuan	104° 32′ 13″	34° 30′ 03″	2166
319	Dingxi	Zhangxian	Shichuan	104° 32′ 09″	34° 30′ 10″	2165
320	Dingxi	Zhangxian	Shichuan	104° 31′ 89″	34° 59′ 30″	2170
321	Dingxi	Zhangxian	Shichuan	104° 28′ 85″	34° 58′ 34″	2289
322	Dingxi	Zhangxian	Shichuan	104° 29′ 51″	34° 57′ 83″	2280
323	Dingxi	Zhangxian	Shichuan	104° 34′ 72″	34° 66′ 23″	2060
324	Dingxi	Zhangxian	Shichuan	104° 34′ 62″	34° 66′ 20″	2062
325	Dingxi	Zhangxian	Shichuan	104° 33′ 39″	34° 64′ 99″	2072
326	Dingxi	Zhangxian	Shichuan	104° 33′ 15″	34° 64′ 83″	2073
327	Dingxi	Zhangxian	Shichuan	104° 33′ 11″	34° 63′ 97″	2090
328	Dingxi	Zhangxian	Shichuan	104° 32′ 14″	34° 60′ 70″	2158
329	Dingxi	Zhangxian	Sancha	104° 29′ 47″	34° 87′ 88″	2097
330	Dingxi	Zhangxian	Chihuqiao	104° 29′ 53″	34° 84′ 15″	2115
331	Dingxi	Zhangxian	Chihuqiao	104° 29′ 65″	34° 84′ 03″	2137
332	Dingxi	Zhangxian	Sancha	104° 36′ 70″	34° 92′ 42″	2302
333	Dingxi	Zhangxian	Yanjing	104° 44′ 41″	34° 81′ 23″	2175

334	Dingxi	Zhangxian	Sancha	104° 38′ 48″	34° 90′ 89″	2177
335	Dingxi	Zhangxian	Sancha	104° 35′ 10″	34° 90′ 77″	2084
336	Dingxi	Zhangxian	Sancha	104° 32′ 15″	34° 92′ 66″	2213
337	Dingxi	Zhangxian	Sancha	104° 32′ 24″	34° 89′ 78″	2109
338	Dingxi	Zhangxian	Yihuqiao	104° 17′ 52″	34° 90′ 24″	2346
339	Dingxi	Zhangxian	Yihuqiao	104° 18′ 75″	34° 89′ 98″	2310
340	Dingxi	Zhangxian	Yihuqiao	104° 24′ 04″	34° 87′ 38″	2150
341	Dingxi	Zhangxian	Yihuqiao	104° 22′ 05″	34° 85′ 66″	2232
342	Dingxi	Zhangxian	Yihuqiao	104° 22′ 55″	34° 85′ 71″	2212
343	Dingxi	Zhangxian	Yihuqiao	104° 24′ 79″	34° 85′ 03″	2347
344	Dingxi	Zhangxian	Yihuqiao	104° 27′ 32″	34° 87′ 61″	2297
345	Dingxi	Zhangxian	Yihuqiao	104° 23′ 98″	34° 53′ 59″	2333
346	Dingxi	Zhangxian	Sizu	104° 35′ 74″	34° 68′ 40″	2224
347	Dingxi	Zhangxian	Sizu	104° 34′ 88″	34° 68′ 46″	2352
348	Dingxi	Zhangxian	Sizu	104° 31′ 82″	34° 69′ 52″	2229
349	Dingxi	Zhangxian	Sizu	104° 31′ 11″	34° 69′ 18″	2229
350	Dingxi	Zhangxian	Sizu	104° 33′ 17″	34° 69′ 65″	2241
351	Dingxi	Zhangxian	Sizu	104° 32′ 71″	34° 72′ 45″	2289
352	Dingxi	Zhangxian	Sizu	104° 32′ 68″	34° 72′ 44″	2314
353	Dingxi	Zhangxian	Sizu	104° 33′ 09″	34° 71′ 17″	2165
354	Dingxi	Zhangxian	Sizu	104° 35′ 35″	34° 72′ 67″	2325
355	Dingxi	Zhangxian	Sizu	104° 36′ 57″	34° 71′ 85″	2210
356	Dingxi	Zhangxian	Sizu	104° 41′ 54″	34° 67′ 50″	2032
357	Dingxi	Zhangxian	Sizu	104° 41′ 50″	34° 67′ 57″	2024
358	Dingxi	Zhangxian	Maquan	104° 40′ 69″	34° 73′ 37″	2289
359	Dingxi	Zhangxian	Maquan	104° 41′ 47″	34° 76′ 12″	2227
360	Dingxi	Zhangxian	Maquan	104° 42′ 31″	34° 76′ 53″	2365
361	Dingxi	Minxian	Zhongzhai	103° 56′ 25″	34° 38′ 05″	2387
362	Dingxi	Minxian	Zhongzhai	103° 58′ 37″	34° 38′ 03″	2379
363	Dingxi	Minxian	Zhongzhai	103° 56′ 43″	34° 39′ 36″	2397
364	Dingxi	Minxian	Zhongzhai	103° 58′ 37″	34° 40′ 11″	2308
365	Dingxi	Minxian	Zhongzhai	103° 58′ 43″	34° 42′ 02″	2528
366	Dingxi	Minxian	Zhongzhai	103° 58′ 38″	34° 42′ 34″	2699
367	Dingxi	Minxian	Zhongzhai	103° 58′ 02″	34° 42′ 34″	2701
368	Dingxi	Minxian	Zhongzhai	103° 58′ 04″	34° 42′ 34″	2685
369	Dingxi	Minxian	Zhongzhai	103° 58′ 16″	34° 42′ 30″	2639
370	Dingxi	Minxian	Zhongzhai	103° 58′ 39″	34° 41′ 34″	2408
371	Dingxi	Minxian	Zhongzhai	104° 03′ 55.62″	34° 41′ 49.03″	2731
372	Dingxi	Minxian	Zhongzhai	104° 03′ 59.17″	34° 41′ 47.52″	2723
373	Dingxi	Minxian	Zhongzhai	104° 02′ 21.62″	34° 40′ 24.77″	2572
374	Dingxi	Minxian	Zhongzhai	104° 02′ 16.59″	34° 40′ 37.94″	2620
375	Dingxi	Minxian	Zhongzhai	104° 00′ 49.23″	34° 39′ 51.22″	2486
376	Dingxi	Minxian	Zhongzhai	104° 00′ 43.71″	34° 40′ 46.95″	2594
377	Dingxi	Minxian	Zhongzhai	104° 02′ 49.72″	34° 37′ 44.87″	2523
378	Dingxi	Minxian	Zhongzhai	104° 03′ 16.81″	34° 37′ 49.68″	2577
379	Dingxi	Minxian	Zhongzhai	104° 03′ 27.17″	34° 37′ 46.50″	2599
380	Dingxi	Minxian	Zhongzhai	104° 03′ 26.58″	34° 37′ 46.51″	2605
381	Dingxi	Minxian	Zhongzhai	104° 02′ 51.26″	34° 37′ 39.45″	2546

382	Dingxi	Minxian	Zhongzhai	104° 04′ 56.87″	34° 37′ 51.35″	2743
383	Dingxi	Minxian	Zhongzhai	104° 05′ 11.38″	34° 37′ 51.75″	2764
384	Dingxi	Minxian	Zhongzhai	104° 02′ 41.85″	34° 37′ 46.83″	2831
385	Dingxi	Minxian	Zhongzhai	104° 01′ 19.15″	34° 38′ 06.56″	2392
386	Dingxi	Minxian	Zhongzhai	104° 01′ 03.98″	34° 38′ 13.04″	2367
387	Dingxi	Minxian	Zhongzhai	104° 02′ 04.67″	34° 39′ 46.34″	2482
388	Dingxi	Minxian	Zhongzhai	104° 02′ 23.11″	34° 40′ 01.26″	2509
389	Dingxi	Minxian	Zhongzhai	104° 02′ 35.35″	34° 40′ 06.89″	2536
390	Dingxi	Minxian	Minyang	104° 03′ 46.13″	34° 27′ 05.52″	2296
391	Dingxi	Minxian	Minyang	104° 03′ 48.72″	34° 27′ 01.67″	2303
392	Dingxi	Minxian	Minyang	104° 03′ 29.63″	34° 26′ 09.55″	2351
393	Dingxi	Minxian	Minyang	104° 03′ 34.05″	34° 26′ 05.27″	2396
394	Dingxi	Minxian	Minyang	104° 02′ 58.04″	34° 27′ 22.09″	2323
395	Dingxi	Minxian	Minyang	104° 02′ 58.76″	34° 27′ 21.25″	2325
396	Dingxi	Minxian	Minyang	104° 01′ 30.22″	34° 24′ 35.78″	2375
397	Dingxi	Minxian	Hetuo	104° 11′ 48″	34° 28′ 07″	2447
398	Dingxi	Minxian	Hetuo	104° 27′ 55″	34° 29′ 00″	2446
399	Dingxi	Minxian	Hetuo	104° 11′ 08″	34° 28′ 22″	2426
400	Dingxi	Minxian	Hetuo	104° 11′ 08″	34° 28′ 20″	2422
401	Dingxi	Minxian	Hetuo	104° 11′ 00″	34° 28′ 20″	2431
402	Dingxi	Minxian	Hetuo	104° 09′ 09″	34° 28′ 43″	2383
403	Dingxi	Minxian	Hetuo	104° 09′ 10″	34° 28′ 45″	2382
404	Dingxi	Minxian	Hetuo	104° 15′ 33″	34° 25′ 45″	2614
405	Dingxi	Minxian	Hetuo	104° 15′ 01″	34° 26′ 31″	2562
406	Dingxi	Minxian	Hetuo	104° 15′ 43″	34° 27′ 08″	2584
407	Dingxi	Minxian	Hetuo	104° 14′ 02″	34° 26′ 53″	2530
408	Dingxi	Minxian	Hetuo	104° 12′ 26″	34° 27′ 49″	2471
409	Dingxi	Minxian	Hetuo	104° 12′ 22″	34° 27′ 49″	2468
410	Dingxi	Minxian	Puma	104° 22′ 55″	34° 32′ 23″	2497
411	Dingxi	Minxian	Puma	104° 22′ 58″	34° 32′ 35″	2476
412	Dingxi	Minxian	Puma	104° 24′ 03″	34° 32′ 59″	2332
413	Dingxi	Minxian	Puma	104° 24′ 10″	34° 32′ 54″	2340
414	Dingxi	Minxian	Puma	104° 25′ 14″	34° 32′ 46″	2471
415	Dingxi	Minxian	Hetuo	104° 16′ 37″	34° 25′ 24″	2651
416	Dingxi	Minxian	Hetuo	104° 16′ 17″	34° 25′ 33″	2639
417	Dingxi	Minxian	Xijiang	104° 00′ 33.84″	34° 32′ 25.87″	2442
418	Dingxi	Minxian	Xijiang	103° 59′ 41.70″	34° 32′ 14.12″	2478
419	Dingxi	Minxian	Xijiang	103° 59′ 03.25″	34° 31′ 05.42″	2676
420	Dingxi	Minxian	Xijiang	103° 58′ 29.32″	34° 33′ 55.57″	2445
421	Dingxi	Minxian	Xijiang	104° 02′ 15.29″	34° 30′ 56.92″	2499
422	Dingxi	Minxian	Xijiang	104° 01′ 28.63″	34° 31′ 55.91″	2453
423	Dingxi	Minxian	Chabu	104° 04′ 10.77″	34° 28′ 06.31″	2378
424	Dingxi	Minxian	Chabu	104° 04′ 49.86″	34° 29′ 06.98″	2325
425	Dingxi	Minxian	Chabu	104° 04′ 42.76″	34° 28′ 13.16″	2256
426	Dingxi	Minxian	Chabu	104° 07′ 30.61″	34° 27′ 14.66″	2224
427	Dingxi	Minxian	Chabu	104° 09′ 36.46″	34° 25′ 37.66″	2551
428	Dingxi	Minxian	Chabu	104° 09′ 34.51″	34° 25′ 36.78″	2554
429	Dingxi	Minxian	Chabu	104° 09′ 48.96″	34° 24′ 10.25″	2649

430	Dingxi	Minxian	Chabu	104° 09′ 50.16″	34° 23′ 37.44″	2677
431	Dingxi	Minxian	Chabu	104° 09′ 48.70″	34° 23′ 38.72″	2679
432	Dingxi	Minxian	Chabu	104° 07′ 49.07″	34° 27′ 10.22″	2468
433	Dingxi	Minxian	Chabu	104° 07′ 53.42″	34° 27′ 12.30″	2488
434	Dingxi	Minxian	Chabu	104° 07′ 11.29″	34° 28′ 06.33″	2358
435	Dingxi	Minxian	Chabu	104° 07′ 27.71″	34° 27′ 21.68″	2420
436	Dingxi	Minxian	Chabu	104° 06′ 51.68″	34° 27′ 41.89″	2448
437	Dingxi	Minxian	Chabu	104° 08′ 34.44″	34° 29′ 37.53″	2458
438	Dingxi	Minxian	Weixin	103° 52′ 49.00″	34° 39′ 51.00″	2259
439	Dingxi	Minxian	Weixin	103° 53′ 27.21″	34° 37′ 32.62″	2403
440	Dingxi	Minxian	Weixin	103° 53′ 10.97″	34° 37′ 20.99″	2495
441	Dingxi	Minxian	Weixin	103° 53′ 14.32″	34° 37′ 03.39″	2443
442	Dingxi	Minxian	Weixin	103° 52′ 44.67″	34° 35′ 50.14″	2562
443	Dingxi	Minxian	Weixin	103° 53′ 14.76″	34° 35′ 52.48″	2549
444	Dingxi	Minxian	Weixin	103° 54′ 22.79″	34° 37′ 51.59″	2451
445	Dingxi	Minxian	Weixin	103° 54′ 33.03″	34° 37′ 44.94″	2510
446	Dingxi	Minxian	Weixin	103° 51′ 49″	34° 38′ 50″	2364
447	Dingxi	Minxian	Meichuan	104° 06′ 43″	34° 36′ 59″	2672
448	Dingxi	Minxian	Meichuan	104° 07′ 23″	34° 37′ 35″	2727
449	Dingxi	Minxian	Meichuan	104° 06′ 46″	34° 36′ 24″	2690
450	Dingxi	Minxian	Meichuan	104° 06′ 43″	34° 36′ 29″	2655
451	Dingxi	Minxian	Meichuan	104° 06′ 38″	34° 36′ 40″	2684
452	Dingxi	Minxian	Meichuan	104° 04′ 26″	34° 34′ 56″	2602
453	Dingxi	Minxian	Meichuan	104° 08′ 10″	34° 33′ 50″	2479
454	Dingxi	Minxian	Meichuan	104° 08′ 45″	34° 35′ 16″	2587
455	Dingxi	Minxian	Meichuan	104° 08′ 57″	34° 35′ 25″	2591
456	Dingxi	Minxian	Meichuan	104° 09′ 15″	34° 36′ 04″	2638
457	Dingxi	Minxian	Meichuan	104° 09′ 30″	34° 32′ 26″	2662
458	Dingxi	Minxian	Meichuan	104° 09′ 10″	34° 32′ 44″	2636
459	Dingxi	Minxian	Meichuan	104° 08′ 22″	34° 31′ 15″	2644
460	Dingxi	Minxian	Meichuan	104° 07′ 43″	34° 30′ 42″	2621
461	Dingxi	Minxian	Meichuan	104° 07′ 00″	34° 31′ 07″	2490
462	Dingxi	Minxian	Meichuan	104° 09′ 30″	34° 30′ 41″	2597
463	Dingxi	Minxian	Meichuan	104° 09′ 33″	34° 30′ 38″	2627
464	Dingxi	Minxian	Weixin	103° 55′ 02.95″	34° 39′ 51.06″	2170
465	Dingxi	Minxian	Weixin	103° 55′ 05.07″	34° 39′ 52.42″	2186
466	Dingxi	Minxian	Weixin	103° 55′ 04.75″	34° 42′ 11.46″	2465
467	Dingxi	Minxian	Weixin	103° 55′ 05.49″	34° 42′ 10.83″	2514
468	Dingxi	Minxian	Weixin	103° 54′ 38.50″	34° 41′ 22.53″	2605
469	Dingxi	Minxian	Xizhai	103° 48′ 52.93″	34° 29′ 24.13″	2364
470	Dingxi	Minxian	Xizhai	103° 48′ 48.48″	34° 29′ 50.24″	2368
471	Dingxi	Minxian	Xizhai	103° 47′ 17.63″	34° 31′ 21.74″	2400
472	Dingxi	Minxian	Lujing	104° 28′ 26.84″	34° 25′ 06.92″	2415
473	Dingxi	Minxian	Lujing	104° 28′ 25.12″	34° 25′ 11.05″	2438
474	Dingxi	Minxian	Lujing	104° 28′ 41.06″	34° 24′ 56.31″	2485
475	Dingxi	Minxian	Lujing	104° 30′ 12.00″	34° 23′ 17.21″	2451
476	Dingxi	Minxian	Lujing	104° 30′ 14.20″	34° 23′ 19.04″	2453
477	Dingxi	Minxian	Lujing	104° 30′ 10.66″	34° 23′ 11.04″	2493

478	Dingxi	Minxian	Lujing	104° 30′ 39.42″	34° 22′ 31.75″	2491
479	Dingxi	Minxian	Lujing	104° 30′ 41.03″	34° 22′ 34.78″	2476
480	Dingxi	Minxian	Lujing	104° 30′ 29.56″	34° 22′ 28.39″	2498
481	Dingxi	Minxian	Lujing	104° 29′ 46.65″	34° 22′ 41.59″	2497
482	Dingxi	Minxian	Lujing	104° 29′ 39.39″	34° 22′ 23.84″	2519
483	Dingxi	Minxian	Lujing	104° 29′ 39.95″	34° 22′ 24.00″	2553
484	Dingxi	Minxian	Lujing	104° 32′ 15.12″	34° 21′ 22.08″	2507
485	Dingxi	Minxian	Lujing	104° 32′ 11.08″	34° 21′ 17.75″	2470
486	Dingxi	Minxian	Lujing	104° 32′ 15.71″	34° 21′ 11.71″	2435
487	Dingxi	Minxian	Lujing	104° 33′ 08.64″	34° 20′ 41.06″	2547
488	Dingxi	Minxian	Lujing	104° 33′ 07.27″	34° 20′ 41.75″	2542
489	Dingxi	Minxian	Lujing	104° 33′ 07.03″	34° 20′ 41.51″	2528
490	Dingxi	Minxian	Lujing	104° 33′ 33.50″	34° 21′ 04.63″	2609
491	Dingxi	Minxian	Lujing	104° 33′ 31.36″	34° 21′ 51.58″	2829
492	Dingxi	Minxian	Lujing	104° 33′ 38.99″	34° 21′ 58.58″	2805
493	Dingxi	Minxian	Lujing	104° 33′ 53.66″	34° 24′ 48.27″	2646
494	Dingxi	Minxian	Lujing	104° 33′ 50.17″	34° 24′ 44.77″	2594
495	Dingxi	Minxian	Lujing	104° 33′ 45.93″	34° 24′ 50.04″	2581
496	Dingxi	Minxian	Lujing	104° 33′ 47.90″	34° 24′ 59.86″	2621
497	Dingxi	Minxian	Lujing	104° 34′ 20.48″	34° 19′ 46.47″	2576
498	Dingxi	Minxian	Lujing	104° 34′ 42.04″	34° 20′ 02.34″	2594
499	Dingxi	Minxian	Lujing	104° 34′ 51.32″	34° 19′ 56.30″	2608
500	Dingxi	Minxian	Lujing	104° 35′ 59.48″	34° 20′ 08.15″	2650
501	Dingxi	Minxian	Lujing	104° 36′ 00.86″	34° 20′ 01.36″	2715
502	Dingxi	Minxian	Lujing	104° 36′ 09.09″	34° 20′ 10.12″	2691
503	Dingxi	Minxian	Lujing	104° 37′ 13.53″	34° 20′ 00.57″	2727
504	Dingxi	Minxian	Lujing	104° 37′ 19.85″	34° 20′ 02.54″	2749
505	Dingxi	Minxian	Lujing	104° 37′ 21.28″	34° 19′ 59.40″	2780
506	Dingxi	Minxian	Lujing	104° 37′ 14.87″	34° 20′ 17.85″	2767
507	Dingxi	Minxian	Lujing	104° 37′ 42.05″	34° 21′ 10.26″	2775
508	Dingxi	Minxian	Lujing	104° 37′ 19.27″	34° 21′ 15.09″	2782
509	Dingxi	Minxian	Lujing	104° 37′ 20.04″	34° 21′ 13.23″	2789
510	Dingxi	Minxian	Lujing	104° 36′ 50.86″	34° 20′ 56.18″	2772
511	Dingxi	Minxian	Lujing	104° 32′ 41.90″	34° 19′ 45.29″	2590
512	Dingxi	Minxian	Lujing	104° 32′ 35.33″	34° 19′ 46.10″	2592
513	Dingxi	Minxian	Lujing	104° 32′ 37.94″	34° 19′ 46.26″	2588
514	Dingxi	Minxian	Lujing	104° 31′ 51.52″	34° 18′ 55.68″	2595
515	Dingxi	Minxian	Lujing	104° 31′ 46.08″	34° 18′ 51.09″	2585
516	Dingxi	Minxian	Lujing	104° 31′ 52.36″	34° 19′ 12.80″	2575
517	Dingxi	Minxian	Lujing	104° 32′ 01.00″	34° 20′ 05.17″	2561
518	Dingxi	Minxian	Lujing	104° 31′ 59.46″	34° 20′ 09.94″	2574
519	Dingxi	Minxian	Lujing	104° 30′ 32.91″	34° 20′ 06.54″	2572
520	Dingxi	Minxian	Lujing	104° 30′ 32.91″	34° 20′ 22.92″	2586
521	Dingxi	Minxian	Lujing	104° 30′ 24.39″	34° 20′ 24.56″	2594
522	Dingxi	Minxian	Lujing	104° 30′ 27.59″	34° 20′ 25.18″	2586
523	Dingxi	Minxian	Lujing	104° 29′ 07.78″	34° 19′ 02.48″	2686
524	Dingxi	Minxian	Lujing	104° 29′ 07.30″	34° 18′ 59.29″	2693
525	Dingxi	Minxian	Lujing	104° 28′ 57.34″	34° 18′ 58.85″	2695

526	Dingxi	Minxian	Lujing	104° 28′ 55.81″	34° 17′ 00.26″	2697
527	Dingxi	Minxian	Lujing	104° 28′ 13.95″	34° 18′ 21.64″	2673
528	Dingxi	Minxian	Lujing	104° 28′ 14.97″	34° 18′ 23.03″	2670
529	Dingxi	Minxian	Lujing	104° 28′ 22.15″	34° 18′ 16.41″	2657
530	Dingxi	Minxian	Lujing	104° 28′ 21.87″	34° 18′ 00.94″	2676
531	Dingxi	Minxian	Lujing	104° 27′ 38.05″	34° 20′ 12.61″	2702
532	Dingxi	Minxian	Lujing	104° 27′ 41.05″	34° 21′ 10.52″	2720
533	Dingxi	Minxian	Lujing	104° 27′ 42.49″	34° 20′ 12.25″	2716
534	Dingxi	Minxian	Lujing	104° 27′ 42.75″	34° 20′ 12.71″	2713
535	Dingxi	Minxian	Lujing	104° 27′ 02.59″	34° 20′ 11.26″	2724
536	Dingxi	Minxian	Lujing	104° 26′ 51.75″	34° 20′ 17.81″	2690
537	Dingxi	Minxian	Lujing	104° 26′ 50.40″	34° 20′ 18.94″	2697
538	Dingxi	Minxian	Lujing	104° 28′ 48.14″	34° 19′ 50.57″	2639
539	Dingxi	Minxian	Lujing	104° 29′ 04.49″	34° 19′ 52.90″	2628
540	Dingxi	Minxian	Lujing	104° 30′ 66.41″	34° 19′ 21.63″	2575
541	Dingxi	Minxian	Lujing	104° 33′ 04.40″	34° 19′ 21.00″	2577
542	Dingxi	Minxian	Lujing	104° 34′ 02.91″	34° 19′ 41.15″	2580
543	Dingxi	Minxian	Lujing	104° 34′ 47.47″	34° 18′ 11.09″	2608
544	Dingxi	Minxian	Lujing	104° 34′ 49.34″	34° 18′ 58.04″	2584
545	Dingxi	Minxian	Puma	104° 30′ 02.03″	34° 29′ 23.33″	2670
546	Dingxi	Minxian	Puma	104° 20′ 10.96″	34° 29′ 26.72″	2675
547	Dingxi	Minxian	Puma	104° 20′ 34.84″	34° 29′ 15.41″	2726
548	Dingxi	Minxian	Puma	104° 23′ 14.69″	34° 29′ 19.32″	2535
549	Dingxi	Minxian	Puma	104° 26′ 25.55″	34° 29′ 25.52″	2512
550	Dingxi	Minxian	Puma	104° 23′ 29.12″	34° 29′ 25.45″	2452
551	Dingxi	Minxian	Puma	104° 24′ 01.58″	34° 30′ 55.03″	2427
552	Dingxi	Minxian	Puma	104° 24′ 05.41″	34° 30′ 59.66″	2382
553	Dingxi	Minxian	Puma	104° 24′ 12.39″	34° 31′ 02.93″	2392
554	Dingxi	Minxian	Puma	104° 24′ 40.40″	34° 31′ 53.39″	2404
555	Dingxi	Minxian	Puma	104° 24′ 47.98″	34° 32′ 01.84″	2386
556	Dingxi	Minxian	Puma	104° 28′ 26.26″	34° 28′ 15.59″	2409
557	Dingxi	Minxian	Puma	104° 29′ 11.24″	34° 27′ 57.66″	2367
558	Dingxi	Minxian	Puma	104° 28′ 50.82″	34° 27′ 48.91″	2430
559	Dingxi	Minxian	Puma	104° 27′ 06.97″	34° 27′ 45.21″	2448
560	Dingxi	Minxian	Puma	104° 27′ 05.32″	34° 27′ 43.35″	2465
561	Dingxi	Minxian	Puma	104° 27′ 23.41″	34° 27′ 49.74″	2464
562	Dingxi	Minxian	Puma	104° 27′ 35.39″	34° 27′ 44.64″	2455
563	Dingxi	Minxian	Puma	104° 27′ 41.06″	34° 25′ 22.05″	2437
564	Dingxi	Minxian	Puma	104° 28′ 03.05″	34° 25′ 17.74″	2445
565	Dingxi	Minxian	Puma	104° 28′ 06.73″	34° 25′ 12.39″	2432
566	Dingxi	Minxian	Puma	104° 25′ 57.05″	34° 27′ 02.28″	2454
567	Dingxi	Minxian	Puma	104° 25′ 52.05″	34° 27′ 14.52″	2485
568	Dingxi	Minxian	Puma	104° 25′ 59.04″	34° 27′ 12.83″	2487
569	Dingxi	Minxian	Puma	104° 25′ 55.68″	34° 27′ 22.36″	2487
570	Dingxi	Minxian	Puma	104° 28′ 03″	34° 30′ 59″	2570
571	Dingxi	Minxian	Puma	104° 27′ 58″	34° 30′ 59″	2562
572	Dingxi	Minxian	Puma	104° 27′ 56″	34° 31′ 02″	2590
573	Dingxi	Minxian	Puma	104° 28′ 11″	34° 30′ 52″	2562

574	Dingxi	Minxian	Puma	104° 29′ 13″	34° 30′ 33″	2466
575	Dingxi	Minxian	Puma	104° 29′ 43″	34° 30′ 19″	2463
576	Dingxi	Minxian	Puma	104° 30′ 29″	34° 29′ 36″	2461
577	Dingxi	Minxian	Puma	104° 30′ 31″	34° 29′ 33″	2454
578	Dingxi	Minxian	Puma	104° 30′ 38″	34° 29′ 29″	2458
579	Dingxi	Minxian	Puma	104° 30′ 40″	34° 29′ 32″	2418
580	Dingxi	Minxian	Puma	104° 30′ 50″	34° 28′ 32″	2519
581	Dingxi	Minxian	Puma	104° 30′ 50″	34° 28′ 31″	2515
582	Dingxi	Minxian	Puma	104° 30′ 52″	34° 28′ 31″	2505
583	Dingxi	Minxian	Puma	104° 30′ 57″	34° 29′ 24″	2409
584	Dingxi	Minxian	Puma	104° 29′ 25″	34° 30′ 54″	2548
585	Dingxi	Minxian	Puma	104° 29′ 59″	34° 31′ 25″	2615
586	Dingxi	Minxian	Puma	104° 29′ 57″	34° 31′ 21″	2600
587	Dingxi	Minxian	Puma	104° 29′ 59″	34° 31′ 22″	2628
588	Dingxi	Minxian	Puma	104° 29′ 53″	34° 31′ 09″	2580
589	Dingxi	Minxian	Puma	104° 30′ 56″	34° 30′ 10″	2422
590	Dingxi	Minxian	Puma	104° 30′ 53″	34° 30′ 12″	2426
591	Dingxi	Minxian	Puma	104° 30′ 56″	34° 30′ 37″	2522
592	Dingxi	Minxian	Puma	104° 31′ 12″	34° 30′ 37″	2526
593	Dingxi	Minxian	Puma	104° 32′ 30″	34° 30′ 25″	2443
594	Dingxi	Minxian	Puma	104° 31′ 37″	34° 30′ 39″	2471
595	Dingxi	Minxian	Puma	104° 25′ 35″	34° 31′ 50″	2407
596	Dingxi	Minxian	Puma	104° 26′ 51″	34° 30′ 32″	2565
597	Dingxi	Minxian	Puma	104° 27′ 05″	34° 30′ 18″	2524
598	Dingxi	Minxian	Puma	104° 45′ 10″	34° 50′ 19″	2568
599	Dingxi	Minxian	Puma	104° 27′ 52″	34° 28′ 58″	2460
600	Dingxi	Minxian	Puma	104° 27′ 23″	34° 26′ 56″	2406
601	Dingxi	Minxian	Puma	104° 21′ 17″	34° 35′ 14″	2330
602	Dingxi	Minxian	Puma	104° 21′ 22″	34° 35′ 12″	2329
603	Dingxi	Minxian	Puma	104° 21′ 17″	34° 35′ 11″	2321
604	Dingxi	Minxian	Puma	104° 21′ 15″	34° 35′ 10″	2301
605	Dingxi	Minxian	Puma	104° 21′ 26″	34° 34′ 24″	2252
606	Dingxi	Minxian	Puma	104° 22′ 13″	34° 34′ 03″	2291
607	Dingxi	Minxian	Puma	104° 22′ 26″	34° 33′ 54″	2296
608	Dingxi	Minxian	Puma	104° 22′ 42″	34° 33′ 48″	2296
609	Dingxi	Minxian	Puma	104° 23′ 03″	34° 33′ 44″	2303
610	Dingxi	Minxian	Puma	104° 22′ 58″	34° 35′ 49″	2411
611	Dingxi	Minxian	Puma	104° 23′ 30″	34° 33′ 17″	2321
612	Dingxi	Minxian	Puma	104° 23′ 01″	34° 32′ 44″	2445
613	Dingxi	Minxian	Puma	104° 22′ 57″	34° 32′ 23″	2495
614	Dingxi	Minxian	Puma	104° 26′ 26″	34° 26′ 28″	2443
615	Dingxi	Minxian	Puma	104° 25′ 58″	34° 25′ 56″	2457
616	Dingxi	Minxian	Puma	104° 25′ 52″	34° 25′ 41″	2452
617	Dingxi	Minxian	Puma	104° 25′ 45″	34° 25′ 33″	2467
618	Dingxi	Minxian	Shengdu	104° 25′ 26″	34° 25′ 00″	2472
619	Dingxi	Minxian	Shengdu	104° 25′ 29″	34° 25′ 01″	2471
620	Dingxi	Minxian	Shengdu	104° 25′ 28″	34° 24′ 59″	2465
621	Dingxi	Minxian	Puma	104° 25′ 28″	34° 24′ 59″	2465

622	Dingxi	Minxian	Shengdu	104° 19′ 14.24″	34° 24′ 57.76″	2782
623	Dingxi	Minxian	Shengdu	104° 19′ 10.88″	34° 25′ 00.35″	2785
624	Dingxi	Minxian	Shengdu	104° 19′ 21.89″	34° 24′ 59.63″	2728
625	Dingxi	Minxian	Shengdu	104° 20′ 58.69″	34° 24′ 24.22″	2659
626	Dingxi	Minxian	Shengdu	104° 21′ 00.07″	34° 24′ 22.24″	2677
627	Dingxi	Minxian	Shengdu	104° 20′ 58.39″	34° 24′ 27.80″	2639
628	Dingxi	Minxian	Shengdu	104° 20′ 47.27″	34° 24′ 20.86″	2646
629	Dingxi	Minxian	Shengdu	104° 21′ 40.67″	34° 24′ 20.36″	2598
630	Dingxi	Minxian	Shengdu	104° 21′ 35.11″	34° 24′ 18.63″	2605
631	Dingxi	Minxian	Shengdu	104° 21′ 35.08″	34° 24′ 23.53″	2608
632	Dingxi	Minxian	Shengdu	104° 22′ 31.87″	34° 24′ 30.37″	2606
633	Dingxi	Minxian	Shengdu	104° 22′ 36.26″	34° 24′ 31.10″	2611
634	Dingxi	Minxian	Shengdu	104° 22′ 28.92″	34° 24′ 45.94″	2622
635	Dingxi	Minxian	Shengdu	104° 22′ 17.51″	34° 23′ 34.79″	2609
636	Dingxi	Minxian	Shengdu	104° 22′ 04.51″	34° 22′ 56.91″	2642
637	Dingxi	Minxian	Shengdu	104° 23′ 12.25″	34° 24′ 09.23″	2556
638	Dingxi	Minxian	Shengdu	104° 25′ 55.83″	34° 24′ 23.63″	2568
639	Dingxi	Minxian	Shengdu	104° 25′ 56.16″	34° 24′ 25.81″	2575
640	Dingxi	Minxian	Shengdu	104° 24′ 42.38″	34° 24′ 08.25″	2584
641	Dingxi	Minxian	Shengdu	104° 24′ 41.51″	34° 24′ 12.10″	2528
642	Dingxi	Minxian	Shengdu	104° 24′ 44.35″	34° 24′ 13.62″	2522
643	Dingxi	Minxian	Shengdu	104° 24′ 06.14″	34° 22′ 27.20″	2574
644	Dingxi	Minxian	Shengdu	104° 24′ 06.73″	34° 21′ 55.50″	2577
645	Dingxi	Minxian	Shengdu	104° 24′ 30.49″	34° 21′ 33.38″	2604
646	Dingxi	Minxian	Shengdu	104° 26′ 07.49″	34° 23′ 30.41″	2578
647	Dingxi	Minxian	Shengdu	104° 26′ 07.49″	34° 22′ 54.82″	2632
648	Dingxi	Minxian	Shengdu	104° 26′ 34.63″	34° 22′ 56.55″	2619
649	Dingxi	Minxian	Mawu	104° 51′ 40.06″	34° 24′ 30.03″	2119
650	Dingxi	Minxian	Mawu	104° 54′ 50.28″	34° 25′ 25.18″	2054
651	Dingxi	Minxian	Mawu	104° 56′ 33.09″	34° 25′ 23.05″	2071
652	Dingxi	Minxian	Mawu	104° 57′ 03.96″	34° 24′ 30.95″	2065
653	Dingxi	Minxian	锁龙	104° 44′ 36.71″	34° 19′ 30.14″	2336
654	Dingxi	Minxian	锁龙	104° 46′ 19.64″	34° 23′ 43.72″	2556
655	Dingxi	Minxian	Mawu	104° 47′ 41.93″	34° 21′ 37.58″	2316
656	Dingxi	Minxian	Mawu	104° 47′ 49.13″	34° 21′ 29.50″	2386
657	Dingxi	Minxian	Mawu	104° 49′ 10.99″	34° 20′ 37.36″	2182
658	Dingxi	Minxian	Mawu	104° 49′ 28.52″	34° 23′ 13.13″	2338
659	Dingxi	Minxian	Mawu	104° 51′ 20.10″	34° 24′ 39.64″	2167
660	Dingxi	Minxian	Mawu	104° 50′ 43.69″	34° 25′ 35.47″	2238
661	Dingxi	Minxian	Sigou	104° 04′ 33.08″	34° 24′ 28.26″	2348
662	Dingxi	Minxian	Sigou	104° 05′ 32.86″	34° 23′ 39.65″	2460
663	Dingxi	Minxian	Sigou	104° 05′ 35.96″	34° 23′ 37.89″	2459
664	Dingxi	Minxian	Sigou	104° 06′ 39.64″	34° 23′ 18.78″	2526
665	Dingxi	Minxian	Sigou	104° 07′ 34.07″	34° 22′ 37.07″	2607
666	Dingxi	Minxian	Sigou	104° 07′ 32.93″	34° 22′ 37.32″	2603
667	Dingxi	Minxian	Sigou	104° 06′ 29.84″	34° 23′ 51.58″	2579
668	Dingxi	Minxian	Sigou	104° 04′ 36.28″	34° 22′ 57.15″	2371
669	Dingxi	Minxian	Sigou	104° 04′ 44.71″	34° 22′ 15.67″	2382

670	Dingxi	Minxian	Sigou	104° 04′ 46.33″	34° 21′ 40.04″	2389
671	Dingxi	Minxian	Sigou	104° 04′ 46.26″	34° 21′ 36.61″	2387
672	Dingxi	Minxian	Sigou	104° 04′ 49.48″	34° 21′ 41.01″	2395
673	Dingxi	Minxian	Sigou	104° 06′ 29.45″	34° 21′ 23.81″	2468
674	Dingxi	Minxian	Sigou	104° 07′ 00.68″	34° 21′ 40.24″	2541
675	Dingxi	Minxian	Sigou	104° 07′ 00.70″	34° 21′ 40.89″	2513
676	Dingxi	Minxian	Sigou	104° 05′ 17.55″	34° 20′ 01.23″	2422
677	Dingxi	Minxian	Sigou	104° 05′ 15.79″	34° 20′ 01.76″	2424
678	Dingxi	Minxian	Sigou	104° 05′ 19.43″	34° 20′ 14.15″	2431
679	Dingxi	Minxian	Sigou	104° 03′ 51.61″	104° 19′ 15.02″	2454
680	Dingxi	Minxian	Sigou	104° 28′ 28.55″	34° 17′ 56.75″	2505
681	Dingxi	Minxian	Sigou	104° 02′ 20.42″	34° 17′ 32.63″	2513
682	Dingxi	Minxian	Sigou	104° 01′ 01.79″	34° 17′ 37.78″	2557
683	Dingxi	Minxian	Sigou	104° 01′ 24.03″	34° 17′ 46.10″	2567
684	Dingxi	Minxian	Sigou	104° 01′ 25.68″	34° 17′ 44.97″	2558
685	Dingxi	Minxian	Mazichuan	104° 04′ 21.07″	34° 18′ 01.99″	2489
686	Dingxi	Minxian	Mazichuan	104° 04′ 19.40″	34° 18′ 00.29″	2486
687	Dingxi	Minxian	Mazichuan	104° 03′ 05.71″	34° 16′ 56.15″	2608
688	Dingxi	Minxian	Mazichuan	104° 03′ 02.24″	34° 16′ 56.28″	2536
689	Dingxi	Minxian	Mazichuan	104° 02′ 52.52″	34° 15′ 57.98″	2572
690	Dingxi	Minxian	Mazichuan	104° 02′ 51.86″	34° 15′ 56.06″	2571
691	Dingxi	Minxian	Mazichuan	104° 04′ 58.17″	34° 18′ 01.78″	2467
692	Dingxi	Minxian	Mazichuan	104° 05′ 00.11″	34° 18′ 03.20″	2462
693	Dingxi	Minxian	Mazichuan	104° 05′ 29.66″	34° 17′ 16.11″	2475
694	Dingxi	Minxian	Mazichuan	104° 05′ 27.27″	34° 17′ 17.42″	2475
695	Dingxi	Minxian	Mazichuan	104° 04′ 50.16″	34° 15′ 33.59″	2555
696	Dingxi	Minxian	Shili	103° 58′ 08.71″	34° 25′ 30.16″	2328
697	Dingxi	Minxian	Shili	104° 00′ 38.05″	34° 23′ 53.95″	2394
698	Dingxi	Minxian	Shili	104° 00′ 40.60″	34° 23′ 47.32″	2392
699	Dingxi	Minxian	Shili	104° 00′ 38.47″	34° 23′ 48.79″	2419
700	Dingxi	Minxian	Shili	104° 00′ 24.55″	34° 25′ 15.06″	2398
701	Dingxi	Minxian	Shili	104° 01′ 13.42″	34° 25′ 20.50″	2379
702	Dingxi	Minxian	Mazichuan	104° 06′ 23.35″	34° 17′ 05.20″	2509
703	Dingxi	Minxian	Mazichuan	104° 06′ 21.47″	34° 17′ 03.07″	2505
704	Dingxi	Minxian	Mazichuan	104° 06′ 19.14″	34° 17′ 04.73″	2501
705	Dingxi	Minxian	Mazichuan	104° 07′ 03.21″	34° 17′ 03.54″	2534
706	Dingxi	Minxian	Mazichuan	104° 07′ 04.19″	34° 17′ 02.82″	2544
707	Dingxi	Minxian	Mazichuan	104° 07′ 05.43″	34° 16′ 28.59″	2544
708	Dingxi	Minxian	Mazichuan	104° 07′ 03.34″	34° 16′ 30.10″	2529
709	Dingxi	Minxian	Mazichuan	104° 07′ 06.42″	34° 16′ 29.13″	2534
710	Dingxi	Minxian	Shili	103° 57′ 07.57″	34° 25′ 13.72″	2331
711	Dingxi	Minxian	Shili	103° 57′ 56.36″	34° 25′ 26.12″	2324
712	Dingxi	Minxian	Shili	103° 59′ 10.25″	34° 25′ 31.43″	2323
713	Dingxi	Minxian	Shili	103° 53′ 03.35″	34° 27′ 07.09″	2355
714	Dingxi	Minxian	Shili	103° 53′ 03.95″	34° 27′ 06.48″	2358
715	Dingxi	Minxian	Shili	103° 53′ 05.52″	34° 27′ 50.04″	2357
716	Dingxi	Minxian	Shili	103° 54′ 06.24″	34° 26′ 49.32″	2349
717	Dingxi	Minxian	Shili	103° 53′ 44.24″	34° 27′ 00.13″	2352

718	Dingxi	Minxian	Shili	103° 54′ 18.58″	34° 26′ 47.77″	2344
719	Dingxi	Minxian	Shili	103° 55′ 19.30″	34° 25′ 43.08″	2340
720	Dingxi	Minxian	Shili	103° 55′ 20.85″	34° 25′ 42.93″	2340
721	Dingxi	Minxian	Shili	103° 56′ 08.82″	34° 25′ 30.21″	2332
722	Dingxi	Minxian	Shili	103° 56′ 04.90″	34° 25′ 28.90″	2327
723	Dingxi	Minxian	Shili	103° 57′ 10.89″	34° 25′ 12.43″	2330
724	Dingxi	Minxian	Xizhai	103° 46′ 13.38″	34° 28′ 57.75″	2388
725	Dingxi	Minxian	Shili	103° 51′ 56.85″	34° 28′ 01.43″	2367
726	Dingxi	Minxian	Zhongzhai	103° 57′ 21.60″	34° 39′ 13.61″	2237
727	Dingxi	Minxian	Hetuo	104° 14′ 16.33″	34° 25′ 09.54″	2655
728	Dingxi	Minxian	Hetuo	104° 12′ 40.36″	34° 25′ 34.70″	2567
729	Dingxi	Minxian	Hetuo	104° 14′ 11.53″	34° 25′ 09.09″	2690
730	Dingxi	Minxian	Hetuo	104° 14′ 16.91″	34° 25′ 09.13″	2662
731	Dingxi	Minxian	Hetuo	104° 12′ 08.75″	34° 30′ 59.86″	2550
732	Dingxi	Minxian	Hetuo	104° 12′ 04.69″	34° 31′ 01.96″	2567
733	Dingxi	Minxian	Hetuo	104° 12′ 03.73″	34° 31′ 01.44″	2558
734	Dingxi	Minxian	Hetuo	104° 12′ 19.39″	34° 32′ 06.17″	2658
735	Dingxi	Minxian	Hetuo	104° 12′ 18.44″	34° 32′ 07.23″	2650
736	Dingxi	Minxian	Hetuo	104° 11′ 29.98″	34° 31′ 51.90″	2695
737	Dingxi	Minxian	Hetuo	104° 11′ 48.01″	34° 30′ 02.53″	2480
738	Dingxi	Minxian	Hetuo	104° 11′ 54.84″	34° 30′ 04.44″	2512
739	Dingxi	Minxian	Hetuo	104° 13′ 10.73″	34° 31′ 07.64″	2508
740	Dingxi	Minxian	Hetuo	104° 13′ 10.67″	34° 31′ 03.08″	2591
741	Dingxi	Minxian	Hetuo	104° 11′ 35.07″	34° 27′ 15.34″	2472
742	Dingxi	Minxian	Hetuo	104° 10′ 42.55″	34° 29′ 38.99″	2422
743	Dingxi	Minxian	Hetuo	104° 10′ 48.60″	34° 29′ 44.22″	2450
744	Dingxi	Minxian	Hetuo	104° 10′ 26.29″	34° 30′ 13.71″	2654
745	Dingxi	Minxian	Hetuo	104° 10′ 22.92″	34° 30′ 14.26″	2659
746	Dingxi	Minxian	Hetuo	104° 12′ 52.56″	34° 27′ 26.04″	2456
747	Dingxi	Minxian	Hetuo	104° 13′ 22.82″	34° 28′ 07.09″	2560
748	Dingxi	Minxian	Hetuo	104° 12′ 38.54″	34° 28′ 41.93″	2574
749	Dingxi	Minxian	Hetuo	104° 12′ 37.48″	34° 28′ 41.16″	2567
750	Dingxi	Minxian	Hetuo	104° 11′ 33.90″	34° 27′ 19.04″	2482
751	Dingxi	Minxian	Shili	103° 58′ 52.00″	34° 24′ 30.00″	2425
752	Dingxi	Minxian	Shili	103° 59′ 36.00″	34° 23′ 41.00″	2406
753	Dingxi	Minxian	Shili	103° 59′ 25.00″	34° 23′ 56.00″	2417
754	Dingxi	Minxian	Weixin	103° 51′ 39.00″	34° 37′ 48.00″	2436
755	Dingxi	Minxian	Shili	103° 57′ 58.00″	34° 25′ 07.00″	2335
756	Dingxi	Minxian	Shili	103° 59′ 09.00″	34° 23′ 35.00″	2494
757	Dingxi	Minxian	Shili	103° 58′ 25.00″	34° 23′ 05.00″	2494
758	Dingxi	Minxian	Shili	103° 58′ 25.00″	34° 24′ 25.00″	2476
759	Dingxi	Minxian	Xijiang	103° 57′ 48.74〃	34° 34′ 30.47〃	2461
760	Dingxi	Minxian	Xijiang	103° 57′ 27.07〃	34° 35′ 43.75〃	2429
761	Dingxi	Minxian	Xijiang	103° 56′ 08.07〃	34° 35′ 38.45〃	2409
762	Dingxi	Minxian	Xijiang	103° 56′ 04.64〃	34° 35′ 36.71〃	2411
763	Dingxi	Minxian	Xijiang	103° 55′ 29.37〃	34° 33′ 09.63〃	2652
764	Dingxi	Minxian	Xijiang	103° 56′ 23.63〃	34° 36′ 40.22〃	2435
765	Dingxi	Minxian	Xijiang	103° 58′ 18.24〃	34° 34′ 56.24〃	2475

766	Dingxi	Minxian	Xijiang	103° 58′ 35.24″	34° 35′ 28.89″	2339
767	Dingxi	Minxian	Qinxu	103° 51′ 26.42″	34° 23′ 12.40″	2679
768	Dingxi	Minxian	Qinxu	103° 50′ 07.04″	34° 22′ 54.64″	2695
769	Dingxi	Minxian	Qinxu	103° 53′ 45.43″	34° 22′ 56.30″	2611
770	Dingxi	Minxian	Qinxu	103° 54′ 11.32″	34° 23′ 02.06″	2587
771	Dingxi	Minxian	Qinxu	103° 56′ 13″	34° 22′ 45″	2544
772	Dingxi	Minxian	Qinxu	103° 55′ 33″	34° 21′ 39″	2574
773	Dingxi	Minxian	Qinxu	103° 58′ 48″	34° 21′ 14″	2571
774	Dingxi	Minxian	Qinxu	103° 59′ 46″	34° 21′ 36″	2458
775	Dingxi	Minxian	Qinxu	103° 59′ 47″	34° 21′ 35″	2462
776	Dingxi	Minxian	Qinxu	104° 00′ 09″	34° 21′ 54″	2440
777	Dingxi	Minxian	Qinxu	104° 01′ 03″	34° 22′ 50″	2424
778	Dingxi	Minxian	Qinxu	104° 01′ 15″	34° 22′ 28″	2415
779	Dingxi	Minxian	Qinxu	104° 01′ 27″	34° 21′ 47″	2443
780	Dingxi	Minxian	Qinxu	104° 02′ 18″	34° 21′ 19″	2498
781	Dingxi	Minxian	Qinxu	104° 02′ 12″	34° 22′ 48″	2394
782	Dingxi	Minxian	Qinxu	104° 02′ 46″	34° 23′ 22″	2371
783	Dingxi	Minxian	Qinxu	104° 03′ 08″	34° 23′ 29″	2374
784	Dingxi	Minxian	Qinxu	104° 03′ 04″	34° 23′ 27″	2369
785	Dingxi	Minxian	Qinxu	104° 01′ 30″	34° 23′ 00″	2414
786	Dingxi	Minxian	Qinxu	104° 01′ 59″	34° 23′ 24″	2449
787	Dingxi	Minxian	Qinxu	104° 02′ 32″	34° 23′ 38″	2516
788	Dingxi	Minxian	Qinxu	104° 02′ 15″	34° 24′ 00″	2510
789	Dingxi	Minxian	Qinxu	104° 03′ 22″	34° 24′ 23″	2371
790	Dingxi	Minxian	Qinxu	104° 03′ 17″	34° 24′ 31″	2411
791	Dingxi	Minxian	Suolong	104° 41′ 04″	34° 22′ 50″	2607
792	Dingxi	Minxian	Suolong	104° 45′ 07″	34° 25′ 12″	2331
793	Dingxi	Minxian	Suolong	104° 45′ 07″	34° 25′ 12″	2331
794	Dingxi	Minxian	Suolong	104° 42′ 12″	34° 23′ 49″	2524
795	Dingxi	Minxian	Suolong	104° 42′ 06″	34° 24′ 21″	2160
796	Dingxi	Minxian	Suolong	104° 42′ 09″	34° 24′ 21″	2494
797	Dingxi	Minxian	Suolong	104° 42′ 07″	34° 25′ 34″	2646
798	Dingxi	Minxian	Suolong	104° 41′ 15″	34° 25′ 45″	2680
799	Dingxi	Minxian	Suolong	104° 41′ 16″	34° 25′ 46″	2663
800	Dingxi	Minxian	Suolong	104° 41′ 48″	34° 26′ 31″	2661
801	Dingxi	Minxian	Suolong	104° 40′ 48″	34° 26′ 24″	2676
802	Dingxi	Minxian	Suolong	104° 40′ 38″	34° 26′ 15″	2676
803	Dingxi	Minxian	Suolong	104° 40′ 23″	34° 24′ 56″	2581
804	Dingxi	Minxian	Suolong	104° 40′ 24″	34° 24′ 53″	2578
805	Dingxi	Minxian	Suolong	104° 41′ 05″	34° 24′ 57″	2588
806	Dingxi	Minxian	Suolong	104° 41′ 05″	34° 24′ 52″	2586
807	Dingxi	Minxian	Suolong	104° 41′ 15″	34° 23′ 46″	2596
808	Dingxi	Minxian	Suolong	104° 41′ 15″	34° 23′ 45″	2593
809	Dingxi	Minxian	Qingshui	103° 99′ 01″	34° 44′ 06″	2282
810	Dingxi	Minxian	Qingshui	103° 95′ 69″	34° 43′ 36″	2276
811	Dingxi	Minxian	Qingshui	103° 95′ 69″	34° 43′ 35″	2286
812	Dingxi	Minxian	Qingshui	103° 94′ 35″	34° 42′ 91″	2283
813	Dingxi	Minxian	Qingshui	103° 93′ 56″	34° 43′ 26″	2296

814	Dingxi	Minxian	Qingshui	103° 91′ 82〃	34° 44′ 69〃	2317
815	Dingxi	Minxian	Qingshui	103° 89′ 27〃	34° 45′ 86〃	2324
816	Dingxi	Minxian	Qingshui	104° 00′ 15〃	34° 27′ 12〃	2383
817	Dingxi	Minxian	Qingshui	104° 00′ 22〃	34° 27′ 14〃	2461
818	Dingxi	Minxian	Qingshui	103° 58′ 57〃	34° 28′ 21〃	2363
819	Dingxi	Minxian	Qingshui	103° 59′ 52〃	34° 29′ 53〃	2526
820	Dingxi	Minxian	Qingshui	103° 57′ 52〃	34° 29′ 20〃	2486
821	Dingxi	Minxian	Qingshui	103° 57′ 58〃	34° 30′ 44〃	2496
822	Dingxi	Minxian	Qingshui	103° 57′ 40〃	34° 31′ 30〃	2519
823	Dingxi	Minxian	Qingshui	103° 59′ 33〃	34° 27′ 14〃	2368
824	Dingxi	Minxian	Qingshui	103° 57′ 50〃	34° 26′ 40〃	2329
825	Dingxi	Minxian	Qingshui	103° 56′ 56〃	34° 27′ 32〃	2425
826	Dingxi	Minxian	Qingshui	103° 56′ 59〃	34° 27′ 41〃	2416
827	Dingxi	Minxian	Qingshui	103° 54′ 59〃	34° 27′ 35〃	2347
828	Dingxi	Minxian	Qingshui	103° 55′ 17〃	34° 27′ 55〃	2343
829	Dingxi	Minxian	Qingshui	103° 55′ 17〃	34° 28′ 16〃	2388
830	Dingxi	Minxian	Qingshui	103° 55′ 24〃	34° 28′ 36〃	2394
831	Dingxi	Minxian	Qingshui	103° 55′ 28〃	34° 28′ 53〃	2498
832	Dingxi	Minxian	Qingshui	103° 53′ 34〃	34° 29′ 33〃	2416
833	Dingxi	Minxian	Qingshui	103° 24′ 32〃	34° 29′ 31〃	2434
834	Dingxi	Minxian	Qingshui	103° 52′ 47〃	34° 31′ 29〃	2533
835	Dingxi	Minxian	Qingshui	103° 52′ 46〃	34° 31′ 32〃	2529
836	Dingxi	Minxian	Qingshui	103° 52′ 13〃	34° 32′ 11〃	2516
837	Dingxi	Minxian	Qingshui	103° 52′ 13〃	34° 32′ 11〃	2566
838	Dingxi	Minxian	Qingshui	103° 52′ 42〃	34° 27′ 42〃	2309
839	Dingxi	Minxian	Qingshui	103° 53′ 32〃	34° 27′ 30〃	2312
840	Dingxi	Minxian	Qingshui	104° 00′ 53〃	34° 28′ 16〃	2448
841	Dingxi	Minxian	Qingshui	104° 00′ 40〃	34° 28′ 29〃	2485
842	Dingxi	Minxian	Qingshui	103° 58′ 33〃	34° 26′ 49〃	2406
843	Dingxi	Minxian	Xizhai	103° 52′ 28〃	34° 28′ 07〃	2346
844	Dingxi	Minxian	Xizhai	103° 51′ 50〃	34° 28′ 35〃	2292
845	Dingxi	Minxian	Meichuan	104° 06′ 38.62″	34° 36′ 59.03″	2634
846	Lanzhou	Yongdeng	Wuyi	103° 08′ 51″	36° 83′ 42″	2514
847	Lanzhou	Yongdeng	Wuyi	103° 19′ 52″	36° 86′ 75″	2259
848	Lanzhou	Yongdeng	Zhongbao	103° 18′ 87″	36° 80′ 21″	2198
849	Tianshui	Qingshui	Qingting	106° 25′ 52.77″	34° 47′ 22.16″	2054
850	Tianshui	Qingshui	Qingting	106° 25′ 39.78″	34° 47′ 29.58″	2020
851	Tianshui	Qingshui	Qingting	106° 25′ 37.32″	34° 47′ 32.26″	2033
852	Tianshui	Wushan	Yanan	104° 54′ 31.26″	34° 29′ 51.25″	2150
853	Tianshui	Wushan	Yanan	104° 53′ 30.91″	34° 29′ 08.68″	2089
854	Tianshui	Wushan	Yanan	104° 52′ 32.09″	34° 28′ 46.35″	2215
855	Tianshui	Wushan	Yanan	104° 51′ 09.79″	34° 27′ 38.40″	2230
856	Tianshui	Wushan	Yanan	104° 51′ 11.84″	34° 27′ 42.84″	2226
857	Tianshui	Wushan	Yanan	104° 56′ 48.84″	34° 27′ 40.44″	2173
858	Tianshui	Wushan	Yanan	104° 56′ 40.49″	34° 29′ 18.29″	2304
859	Tianshui	Wushan	Yanan	104° 56′ 38.23″	34° 29′ 20.27″	2313
860	Tianshui	Wushan	Yanghe	105° 02′ 09.34″	34° 27′ 53.30″	2021
861	Tianshui	Wushan	Yanghe	104° 59′ 51.33″	34° 30′ 16.06″	1903

862	Tianshui	Wushan	Yanghe	104° 58′ 11.71″	34° 29′ 51.34″	2139
863	Tianshui	Wushan	Yanghe	104° 57′ 21.35″	34° 30′ 14.36″	2061
864	Tianshui	Wushan	Yanghe	104° 58′ 01.85″	34° 30′ 21.64″	2119
865	Tianshui	Wushan	Yanghe	104° 58′ 11.68″	34° 30′ 49.41″	2139
866	Tianshui	Wushan	Yanghe	104° 58′ 41.17″	34° 30′ 26.99″	2162
867	Gannan	Zhuoni	Liuling	103° 30′ 57″	34° 37′ 15″	2706
868	Gannan	Zhuoni	Liuling	103° 30′ 10″	34° 39′ 32″	2752
869	Gannan	Zhuoni	Liuling	103° 30′ 10″	34° 39′ 30″	2763
870	Gannan	Zhuoni	Liuling	103° 28′ 52″	34° 37′ 59″	3708
871	Gannan	Zhuoni	Liuling	103° 28′ 53″	34° 57′ 57″	3808
872	Gannan	Zhuoni	Liuling	103° 27′ 51″	34° 35′ 41″	2543
873	Gannan	Zhuoni	Liuling	103° 30′ 59″	34° 35′ 14″	2618
874	Gannan	Zhuoni	Liuling	103° 30′ 09″	34° 35′ 45″	2595
875	Gannan	Zhuoni	Liuling	103° 30′ 50″	34° 35′ 40″	2563
876	Gannan	Zhuoni	Taoyan	103° 50′ 89″	34° 46′ 04″	2367
877	Gannan	Zhuoni	Taoyan	103° 51′ 37″	34° 46′ 11″	2552
878	Gannan	Zhuoni	Taoyan	103° 52′ 05″	34° 45′ 37″	2576
879	Gannan	Zhuoni	Taoyan	103° 53′ 14″	34° 45′ 46″	2782
880	Gannan	Zhuoni	Taoyan	103° 53′ 14″	34° 45′ 44″	2794
881	Gannan	Zhuoni	Zangbawa	103° 55′ 25″	34° 45′ 30″	3908
882	Gannan	Zhuoni	Zangbawa	103° 55′ 35″	34° 45′ 24″	3876
883	Gannan	Zhuoni	Zangbawa	103° 57′ 20″	34° 45′ 48″	3859
884	Gannan	Zhuoni	Zangbawa	103° 57′ 16″	34° 45′ 48″	5854
885	G	Zhuoni	Zangbawa	103° 55′ 17″	34° 46′ 41″	5693
886	Gannan	Zhuoni	Zangbawa	103° 54′ 55″	34° 47′ 10″	5645
887	Gannan	Zhuoni	Zangbawa	103° 54′ 52″	34° 49′ 37″	2486
888	Gannan	Zhuoni	Zangbawa	103° 54′ 28″	34° 49′ 55″	2440
889	Gannan	Zhuoni	Zangbawa	103° 58′ 22″	34° 53′ 54″	2700
890	Gannan	Zhuoni	Zangbawa	103° 58′ 05″	34° 53′ 31″	2645
891	Gannan	Zhuoni	Zhuowa	103° 35′ 51″	34° 58′ 36″	2506
892	Gannan	Zhuoni	Zhuowa	103° 35′ 54″	34° 58′ 31″	2471
893	Gannan	Zhuoni	Zhuowa	103° 34′ 43″	34° 58′ 12″	2548
894	Gannan	Zhuoni	Shengzang	103° 30′ 44″	34° 43′ 20″	2889
895	Gannan	Zhuoni	Shengzang	103° 30′ 35″	34° 43′ 16″	2909
896	Gannan	Zhuoni	Shengzang	103° 27′ 23″	34° 42′ 08″	2855
897	Gannan	Zhuoni	Shengzang	103° 27′ 16″	34° 42′ 21″	2879
898	Gannan	Zhuoni	Shengzang	103° 25′ 41″	34° 43′ 57″	2989
899	Gannan	Zhuoni	Shengzang	103° 27′ 36″	34° 44′ 13″	2912
900	Gannan	Zhuoni	Shengzang	103° 20′ 35″	34° 44′ 36″	2880
901	Gannan	Zhuoni	Keerqing	103° 27′ 32″	34° 40′ 20″	2763
902	Gannan	Zhuoni	Keerqing	103° 25′ 24″	34° 38′ 07″	2823
903	Gannan	Zhuoni	Keerqing	103° 23′ 32″	34° 38′ 18″	2850
904	Gannan	Lingtan	Changchuan	103° 25′ 47″	34° 42′ 74″	2839
905	Gannan	Lingtan	Changchuan	103° 25′ 57″	34° 43′ 21″	2936
906	Gannan	Lingtan	Changchuan	103° 25′ 21″	34° 43′ 24″	2929
907	Gannan	Lingtan	Changchuan	103° 25′ 26″	34° 43′ 21″	2940
908	Gannan	Lingtan	Changchuan	103° 26′ 15″	34° 43′ 16″	2932
909	Gannan	Lingtan	Changchuan	103° 26′ 16″	34° 43′ 22″	2948

910	Gannan	Lingtan	Changchuan	103° 26′ 45″	34° 42′ 53″	2897
911	Gannan	Lingtan	Changchuan	103° 27′ 24″	34° 42′ 28″	2867
912	Gannan	Lingtan	Changchuan	103° 27′ 53″	34° 42′ 10″	2849
913	Gannan	Lingtan	Changchuan	103° 27′ 54″	34° 42′ 05″	2812
914	Gannan	Lingtan	Yangyong	103° 28′ 27″	34° 42′ 29″	2825
915	Gannan	Lingtan	Yangyong	103° 28′ 43″	34° 41′ 57″	2751
916	Gannan	Lingtan	Yangyong	103° 29′ 55″	34° 40′ 48″	2747
917	Gannan	Lingtan	Yangyong	103° 29′ 37″	34° 40′ 38″	2690
918	Gannan	Lingtan	Yangyong	103° 29′ 15″	34° 38′ 36″	2648
919	Gannan	Lingtan	Yangyong	103° 29′ 12″	34° 38′ 40″	2652
920	Gannan	Lingtan	Yangyong	103° 28′ 20″	34° 38′ 17″	2637
921	Gannan	Lingtan	Yangyong	103° 28′ 28″	34° 38′ 26″	2654
922	Gannan	Lingtan	Yangyong	103° 27′ 47″	34° 39′ 26″	2691
923	Gannan	Lingtan	Yangyong	103° 27′ 42″	34° 39′ 31″	2724
924	Gannan	Lingtan	Yangyong	103° 27′ 04″	34° 39′ 03″	2707
925	Gannan	Lingtan	Yangyong	103° 27′ 05″	34° 39′ 05″	2711
926	Gannan	Lingtan	Yangyong	103° 26′ 15″	34° 38′ 42″	2787
927	Gannan	Lingtan	Yangyong	103° 26′ 13″	34° 38′ 41″	2774
928	Gannan	Lingtan	Changchuan	103° 24′ 57″	34° 39′ 23″	2778
929	Gannan	Lingtan	Changchuan	103° 24′ 59″	34° 39′ 24″	2799
930	Gannan	Lingtan	Chengguan	103° 22′ 02″	34° 41′ 07″	2805
931	Gannan	Lingtan	Chengguan	103° 21′ 59″	34° 41′ 09″	2801
932	Gannan	Lingtan	Liushun	103° 33′ 04″	34° 37′ 38″	2644
933	Gannan	Lingtan	Liushun	103° 33′ 04″	34° 37′ 38″	2644
934	Gannan	Lingtan	Liushun	103° 33′ 23″	34° 38′ 57″	2670
935	Gannan	Lingtan	Liushun	103° 33′ 19″	34° 38′ 57″	2677
936	Gannan	Lingtan	Liushun	103° 32′ 37″	34° 39′ 45″	2692
937	Gannan	Lingtan	Liushun	103° 32′ 39″	34° 39′ 45″	2693
938	Gannan	Lingtan	Liushun	103° 32′ 15″	34° 39′ 49″	2712
939	Gannan	Lingtan	Liushun	103° 32′ 18″	34° 39′ 50″	2706
940	Gannan	Lingtan	Liushun	103° 32′ 31″	34° 41′ 03″	2697
941	Gannan	Lingtan	Liushun	103° 32′ 25″	34° 41′ 09″	2764
942	Gannan	Lingtan	Liushun	103° 32′ 41″	34° 40′ 35″	2760
943	Gannan	Lingtan	Liushun	103° 32′ 52″	34° 40′ 25″	2721
944	Gannan	Lingtan	Xingcheng	103° 34′ 44″	34° 40′ 02″	2864
945	Gannan	Lingtan	Xingcheng	103° 35′ 08″	34° 40′ 13″	2715
946	Gannan	Lingtan	Xingcheng	103° 35′ 45″	34° 39′ 53″	2687
947	Gannan	Lingtan	Xingcheng	103° 35′ 42″	34° 39′ 38″	2741
948	Gannan	Lingtan	Xingcheng	103° 35′ 59″	34° 38′ 58″	2761
949	Gannan	Lingtan	Xingcheng	103° 35′ 59″	34° 39′ 01″	2721
950	Gannan	Lingtan	Xingcheng	103° 34′ 42″	34° 40′ 40″	2739
951	Gannan	Lingtan	Xingcheng	103° 34′ 42″	34° 40′ 40″	2732
952	Gannan	Lingtan	Xingcheng	103° 33′ 54″	34° 41′ 17″	2750
953	Gannan	Lingtan	Xingcheng	103° 33′ 52″	34° 41′ 13″	2766
954	Gannan	Lingtan	Xingcheng	103° 33′ 25″	34° 41′ 50″	2862
955	Gannan	Lingtan	Xingcheng	103° 33′ 26″	34° 414′ 45″	2823
956	Gannan	Lingtan	Xingcheng	103° 36′ 03″	34° 40′ 45″	2732
957	Gannan	Lingtan	Xingcheng	103° 35′ 59″	34° 40′ 46″	2735

958	Gannan	Lingtan	Xingcheng	103° 36′ 57″	34° 40′ 06″	2655
959	Gannan	Lingtan	Xingcheng	103° 37′ 43″	34° 40′ 06″	2675
960	Gannan	Lingtan	Xingcheng	103° 38′ 02″	34° 39′ 54″	2607
961	Gannan	Lingtan	Xingcheng	103° 38′ 03″	34° 39′ 50″	2639
962	Gannan	Lingtan	Xingcheng	103° 38′ 19″	34° 40′ 01″	2637
963	Gannan	Lingtan	Xingcheng	103° 38′ 25″	34° 40′ 04″	2622
964	Gannan	Lingtan	Xingcheng	103° 38′ 16″	34° 40′ 22″	2681
965	Gannan	Lingtan	Xingcheng	103° 38′ 18″	34° 40′ 24″	2691
966	Gannan	Lingtan	Xingcheng	103° 39′ 24″	34° 40′ 39″	2670
967	Gannan	Lingtan	Xingcheng	103° 40′ 11″	34° 40′ 50″	2760
968	Gannan	Lingtan	Xingcheng	103° 39′ 19″	34° 40′ 29″	2675
969	Gannan	Lingtan	Xingcheng	103° 39′ 18″	34° 40′ 27″	2696
970	Gannan	Lingtan	Xingcheng	103° 39′ 17″	34° 38′ 43″	2751
971	Gannan	Lingtan	Xingcheng	103° 39′ 27″	34° 38′ 43″	2730
972	Gannan	Lingtan	Xingcheng	103° 37′ 59″	34° 38′ 46″	2585
973	Gannan	Lingtan	Xingcheng	103° 37′ 58″	34° 38′ 43″	2579
974	Gannan	Lingtan	Xingcheng	103° 34′ 42″	34° 39′ 12″	2903
975	Gannan	Lingtan	Xingcheng	103° 34′ 41″	34° 39′ 11″	2878
976	Gannan	Lingtan	Xingcheng	103° 37′ 15″	34° 41′ 21″	2774
977	Gannan	Lingtan	Xingcheng	103° 37′ 17″	34° 41′ 21″	2768
978	Gannan	Lingtan	Shimeng	103° 38′ 41″	34° 43′ 44″	2681
979	Gannan	Lingtan	Shimeng	103° 39′ 53″	34° 43′ 14″	2568
980	Gannan	Lingtan	Shimeng	103° 41′ 01″	34° 43′ 14″	2520
981	Gannan	Lingtan	Shimeng	103° 41′ 41″	34° 43′ 35″	2393
982	Gannan	Lingtan	Shimeng	103° 41′ 54″	34° 43′ 52″	2422
983	Gannan	Lingtan	Shimeng	103° 42′ 40″	34° 44′ 56″	2385
984	Gannan	Lingtan	Shimeng	103° 43′ 36″	34° 45′ 18″	2324
985	Gannan	Lingtan	Shimeng	103° 43′ 53″	34° 45′ 22″	2304
986	Gannan	Lingtan	Shimeng	103° 45′ 19″	34° 45′ 50″	2235
987	Gannan	Lingtan	Shimeng	103° 45′ 15″	34° 45′ 59″	2214
988	Gannan	Lingtan	Shimeng	103° 44′ 10″	34° 46′ 06″	2470
989	Gannan	Lingtan	Shimeng	103° 44′ 19″	34° 46′ 01″	2429
990	Gannan	Lingtan	Shimeng	103° 42′ 36″	34° 45′ 56″	2375
991	Gannan	Lingtan	Shimeng	103° 42′ 46″	34° 45′ 48″	2460
992	Gannan	Lingtan	Shimeng	103° 42′ 24″	34° 43′ 03″	2624
993	Gannan	Lingtan	Shimeng	103° 42′ 24″	34° 43′ 03″	2630
994	Gannan	Lingtan	Shubu	103° 22′ 45″	34° 39′ 41″	2777
995	Gannan	Lingtan	Shubu	103° 23′ 11″	34° 39′ 55″	2777
996	Gannan	Lingtan	Shubu	103° 18′ 31″	34° 43′ 14″	2945
997	Gannan	Lingtan	Shubu	103° 16′ 59″	34° 39′ 08″	2685
998	Gannan	Lingtan	Shubu	103° 16′ 58″	34° 39′ 10″	2674
999	Gannan	Lingtan	Shubu	103° 17′ 20″	34° 37′ 03″	2646
1000	Gannan	Lingtan	Shubu	103° 17′ 44″	34° 39′ 05″	2603
1001	Gannan	Lingtan	Shubu	103° 16′ 55″	34° 36′ 53″	2629
1002	Gannan	Lingtan	Shubu	103° 16′ 19″	34° 37′ 49″	2603
1003	Gannan	Lingtan	Shubu	103° 15′ 49″	34° 38′ 07″	2633
1004	Gannan	Lingtan	Shubu	103° 15′ 52″	34° 38′ 07″	2617
1005	Gannan	Lingtan	Xingcheng	103° 37′ 47″	34° 38′ 11″	2580

1006	Gannan	Lingtan	Xingcheng	103° 37′ 13″	34° 37′ 15″	2548
1007	Gannan	Lingtan	Xingcheng	103° 36′ 44″	34° 36′ 52″	2534
1008	Gannan	Lingtan	Xingcheng	103° 36′ 40″	34° 36′ 49″	2546
1009	Gannan	Lingtan	Sancha	103° 46′ 55″	34° 32′ 57″	2483
1010	Gannan	Lingtan	Sancha	103° 46′ 49″	34° 33′ 01″	2498
1011	Gannan	Lingtan	Sancha	103° 47′ 03″	34° 33′ 18″	2515
1012	Gannan	Lingtan	Sancha	103° 47′ 32″	34° 33′ 33″	2545
1013	Gannan	Lingtan	Sancha	103° 45′ 57″	34° 34′ 39″	2580
1014	Gannan	Lingtan	Sancha	103° 45′ 57″	34° 34′ 42″	2563
1015	Gannan	Lingtan	Sancha	103° 45′ 11″	34° 35′ 14″	2652
1016	Gannan	Lingtan	Sancha	103° 44′ 22″	34° 35′ 05″	2707
1017	Gannan	Lingtan	Sancha	103° 45′ 30″	34° 36′ 19″	2715
1018	Gannan	Lingtan	Sancha	103° 45′ 21″	34° 36′ 50″	2714
1019	Gannan	Lingtan	Taobing	103° 43′ 38″	34° 30′ 19″	2393
1020	Gannan	Lingtan	Taobing	103° 43′ 11″	34° 30′ 46″	2386
1021	Gannan	Lingtan	Taobing	103° 41′ 24″	34° 31′ 51″	2402
1022	Gannan	Lingtan	Taobing	103° 40′ 55″	34° 32′ 34″	2398
1023	Gannan	Lingtan	Taobing	103° 41′ 12″	34° 32′ 50″	2406
1024	Gannan	Lingtan	Dianzi	103° 41′ 27″	34° 39′ 56″	2833
1025	Gannan	Lingtan	Dianzi	103° 41′ 34″	34° 39′ 58″	2810
1026	Gannan	Lingtan	Dianzi	103° 41′ 57″	34° 39′ 56″	2790
1027	Gannan	Lingtan	Dianzi	103° 42′ 07″	34° 39′ 38″	2758
1028	Gannan	Lingtan	Wangqi	103° 44′ 09″	34° 39′ 22″	2718
1029	Gannan	Lingtan	Wangqi	103° 44′ 16″	34° 39′ 22″	2696
1030	Gannan	Lingtan	Wangqi	103° 47′ 18″	34° 39′ 11″	2407
1031	Gannan	Lingtan	Wangqi	103° 47′ 22″	34° 39′ 08″	2440
1032	Gannan	Lingtan	Wangqi	103° 48′ 17″	34° 40′ 26″	2587
1033	Gannan	Lingtan	Wangqi	103° 47′ 54″	34° 40′ 28″	2554
1034	Gannan	Lingtan	Wangqi	103° 49′ 28″	34° 39′ 37″	2326
1035	Gannan	Lingtan	Wangqi	103° 49′ 29″	34° 39′ 36″	2391
1036	Gannan	Lingtan	Wangqi	103° 50′ 00″	34° 39′ 39″	2294
1037	Gannan	Lingtan	Wangqi	103° 48′ 25″	34° 42′ 10″	2331
1038	Gannan	Lingtan	Wangqi	103° 48′ 19″	34° 42′ 15″	2652
1039	Gannan	Lingtan	Wangqi	103° 46′ 52″	34° 42′ 41″	2373
1040	Gannan	Lingtan	Wangqi	103° 46′ 48″	34° 42′ 40″	2390
1041	Gannan	Lingtan	Wangqi	103° 45′ 50″	34° 42′ 29″	2457
1042	Gannan	Lingtan	Wangqi	103° 45′ 32″	34° 42′ 19″	2470
1043	Gannan	Lingtan	Wangqi	103° 50′ 13″	34° 41′ 29″	2406
1044	Gannan	Lingtan	Wangqi	103° 50′ 20″	34° 41′ 33″	2377
1045	Gannan	Lingtan	Wangqi	103° 50′ 29″	34° 40′ 45″	2335
1046	Gannan	Lingtan	Wangqi	103° 50′ 34″	34° 40′ 48″	2301
1047	Gannan	Lingtan	Wangqi	103° 51′ 35″	34° 41′ 08″	2168
1048	Gannan	Lingtan	Wangqi	103° 51′ 27″	34° 40′ 48″	2177
1049	Gannan	Lingtan	Dianzi	103° 41′ 31″	34° 38′ 54″	2774
1050	Gannan	Lingtan	Dianzi	103° 41′ 28″	34° 38′ 53″	2790
1051	Gannan	Lingtan	Dianzi	103° 41′ 27″	34° 37′ 59″	2688
1052	Gannan	Lingtan	Dianzi	103° 41′ 30″	34° 38′ 00″	2710
1053	Gannan	Lingtan	Dianzi	103° 39′ 52″	34° 37′ 35″	2615

1054	Gannan	Lingtan	Guzhan	103° 16′ 11″	34° 42′ 04″	2801
1055	Gannan	Lingtan	Guzhan	103° 15′ 35″	34° 42′ 14″	2809
1056	Gannan	Lingtan	Chengguan	103° 19′ 33″	34° 41′ 44″	2836
1057	Gannan	Lingtan	Chengguan	103° 19′ 28″	34° 41′ 42″	2842
1058	Gannan	Lingtan	Chengguan	103° 22′ 14″	34° 43′ 37″	2835
1059	Gannan	Lingtan	Chengguan	103° 22′ 16″	34° 43′ 08″	2831
1060	Gannan	Lingtan	Chengguan	103° 22′ 27″	34° 40′ 21″	2734
1061	Gannan	Lingtan	Chengguan	103° 23′ 09″	34° 41′ 06″	2847
1062	Gannan	Lingtan	Chengguan	103° 23′ 05″	34° 41′ 03″	2862
1063	Gannan	Lingtan	Chengguan	103° 19′ 27″	34° 41′ 15″	2899
1064	Gannan	Lingtan	Chengguan	103° 19′ 29″	34° 41′ 18″	2912
1065	Gannan	Lingtan	Chengguan	103° 22′ 12″	34° 41′ 38″	2771
1066	Gannan	Lingtan	Chengguan	103° 22′ 14″	34° 41′ 39″	2769
1067	Gannan	Lingtan	Chengguan	103° 21′ 43″	34° 42′ 45″	2800
1068	Gannan	Lingtan	Chengguan	103° 21′ 46″	34° 42′ 45″	2799
1069	Gannan	Lingtan	Chengguan	103° 21′ 46″	34° 42′ 48″	2800
1070	Gannan	Lingtan	Chengguan	103° 21′ 49″	34° 42′ 50″	2806
1071	Gannan	Lingtan	Chengguan	103° 21′ 25″	34° 42′ 42″	2795
1072	Gannan	Lingtan	Chengguan	103° 19′ 27″	34° 41′ 55″	2823
1073	Gannan	Lingtan	Chengguan	103° 20′ 09″	34° 40′ 42″	2883
1074	Gannan	Lingtan	Chengguan	103° 20′ 09″	34° 40′ 42″	2880
1075	Gannan	Lingtan	Chengguan	103° 22′ 47″	34° 40′ 06″	2744
1076	Gannan	Lingtan	Changchuan	103° 24′ 53″	34° 41′ 04″	2790
1077	Gannan	Lingtan	Changchuan	103° 24′ 56″	34° 41′ 06″	2821
1078	Gannan	Lingtan	Changchuan	103° 24′ 41″	34° 40′ 29″	2752
1079	Gannan	Lingtan	Changchuan	103° 24′ 45″	34° 40′ 36″	2784
1080	Gannan	Lingtan	Changchuan	103° 26′ 10″	34° 40′ 54″	2861
1081	Gannan	Lingtan	Changchuan	103° 26′ 11″	34° 40′ 50″	2886
1082	Gannan	Lingtan	Changchuan	103° 26′ 06″	34° 41′ 24″	2833
1083	Gannan	Lingtan	Changchuan	103° 24′ 54″	34° 41′ 28″	2819
1084	Gannan	Lingtan	Dianzi	103° 39′ 14″	34° 26′ 18″	2562
1085	Gannan	Lingtan	Dianzi	103° 40′ 06″	34° 37′ 53″	2653
1086	Gannan	Lingtan	Taobing	103° 38′ 20″	34° 34′ 58″	2519
1087	Gannan	Lingtan	Taobing	103° 38′ 11″	34° 34′ 49″	2514
1088	Gannan	Lingtan	Taobing	103° 37′ 29″	34° 33′ 17″	2443
1089	Gannan	Lingtan	Taobing	103° 36′ 23″	34° 34′ 34″	2461
1090	Gannan	Lingtan	Taobing	103° 35′ 09″	34° 34′ 34″	2478
1091	Gannan	Lingtan	Taobing	103° 36′ 30″	34° 36′ 22″	2513
1092	Gannan	Lingtan	Yangsha	103° 37′ 55″	34° 46′ 36″	2629
1093	Gannan	Lingtan	Yangsha	103° 39′ 23″	34° 48′ 31″	2393
1094	Gannan	Lingtan	Yangsha	103° 41′ 03″	34° 49′ 47″	2310
1095	Gannan	Lingtan	Yangsha	103° 43′ 41″	34° 49′ 54″	2253
1096	Gannan	Lingtan	Yangsha	103° 44′ 21″	34° 52′ 03″	2374
1097	Gannan	Lingtan	Yangsha	103° 42′ 17″	34° 53′ 07″	2552
1098	Gannan	Lingtan	Yangsha	103° 41′ 48″	34° 53′ 20″	2595
1099	Gannan	Lingtan	Yeliguan	103° 28′ 56″	34° 56′ 30″	2362
1100	Gannan	Lingtan	Yeliguan	103° 39′ 30″	34° 56′ 33″	2387
1101	Gannan	Lingtan	Yeliguan	103° 38′ 31″	34° 57′ 47″	2162

1102	Gannan	Lingtan	Yeliguan	103° 36′ 20″	34° 58′ 10″	2408
1103	Gannan	Lingtan	Yeliguan	103° 37′ 36″	34° 57′ 40″	2213
1104	Gannan	Lingtan	Yeliguan	103° 38′ 08″	34° 52′ 70″	2368
1105	Gannan	Lingtan	Yeliguan	103° 38′ 48″	34° 58′ 58″	2231
1106	Gannan	Lingtan	Bajiao	103° 43′ 15″	34° 59′ 54″	2149
1107	Gannan	Lingtan	Bajiao	103° 41′ 31″	34° 59′ 54″	2320
1108	Gannan	Lingtan	Bajiao	103° 43′ 48″	34° 00′ 46″	2315
1109	Gannan	Lingtan	Bajiao	103° 41′ 03″	34° 01′ 17″	2296
1110	Gannan	Lingtan	Bajiao	103° 39′ 47″	34° 02′ 26″	2362
1111	Gannan	Lingtan	Bajiao	103° 39′ 10″	34° 02′ 37″	2427
1112	Gannan	Lingtan	Bajiao	103° 39′ 12″	34° 02′ 34″	2389
1113	Gannan	Lingtan	Bajiao	103° 40′ 03″	34° 02′ 14″	2347
1114	Gannan	Lingtan	Bajiao	103° 41′ 31″	34° 01′ 43″	2254
1115	Gannan	Lingtan	Bajiao	103° 42′ 17″	34° 01′ 23″	2205
1116	Gannan	Zhouqu	Lijie	103° 54′ 43″	34° 54′ 43″	2368
1117	Gannan	Zhouqu	Quwa	103° 59′ 40″	34° 54′ 16″	2288
1118	Gannan	Zhouqu	Quwa	103° 59′ 44″	34° 54′ 17″	2281
1119	Gannan	Zhouqu	Dayu	103° 04′ 54″	34° 46′ 20″	2388
1120	Gannan	Zhouqu	Dayu	103° 04′ 56″	34° 46′ 19″	2393
1121	Gannan	Diebu	Lazikou	103° 57′ 05″	34° 06′ 58″	2291
1122	Gannan	Diebu	Lazikou	103° 57′ 05″	34° 06′ 58″	2291
1123	Gannan	Diebu	Lazikou	103° 59′ 41″	34° 06′ 27″	2526
1124	Gannan	Zhuoni	Nalang	104° 45′ 39″	34° 29′ 29″	2379
1125	Gannan	Zhuoni	Nalang	104° 47′ 05″	34° 30′ 04″	2374
1126	Gannan	Zhuoni	Nalang	103° 40′ 57″	34° 31′ 33″	2448
1127	Gannan	Zhuoni	Nalang	103° 40′ 35″	34° 32′ 21″	2406
1128	Gannan	Zhuoni	Muer	103° 34′ 52″	34° 32′ 25″	2507
1129	Gannan	Zhuoni	Muer	103° 35′ 15″	34° 29′ 51″	2575
1130	Gannan	Zhuoni	Muer	103° 35′ 14″	34° 28′ 01″	2596
1131	Gannan	Zhuoni	Muer	103° 32′ 20″	34° 33′ 37″	2503
1132	Gannan	Zhuoni	Muer	103° 30′ 23″	34° 33′ 47″	2547
1133	Gannan	Zhuoni	Azitan	103° 12′ 26″	34° 39′ 38″	2662
1134	Gannan	Zhuoni	Zhagulu	103° 11′ 09″	34° 39′ 52″	2665
1135	Gannan	Zhuoni	Zhagulu	103° 11′ 13″	34° 39′ 33″	2669
1136	Gannan	Zhuoni	Zhagulu	103° 08′ 55″	34° 39′ 34″	2673
1137	Gannan	Zhuoni	Zhagulu	103° 07′ 52″	34° 38′ 52″	2687
1138	Gannan	Zhuoni	Zhagulu	103° 08′ 18″	34° 39′ 15″	2691
1139	Gannan	Zhuoni	Zhagulu	103° 09′ 57″	34° 39′ 31″	2688
1140	Gannan	Zhuoni	Azitan	103° 14′ 40″	34° 40′ 58″	2997
1141	Gannan	Zhuoni	Azitan	103° 16′ 12″	34° 39′ 57″	3009
1142	Gannan	Zhuoni	Azitan	103° 17′ 03″	34° 42′ 35″	2885
1143	Gannan	Zhuoni	Azitan	103° 18′ 06″	34° 44′ 17″	2878
1144	Gannan	Zhuoni	Azitan	103° 15′ 52″	34° 44′ 15″	2963
1145	Gannan	Zhuoni	Azitan	103° 16′ 31″	34° 38′ 00″	2635
1146	Gannan	Zhuoni	Muer	103° 32′ 14″	34° 36′ 50″	2623
1147	Gannan	Zhuoni	Muer	103° 32′ 36″	34° 38′ 08″	2641
1148	Gannan	Zhuoni	Muer	103° 33′ 33″	34° 37′ 29″	2692
1149	Gannan	Zhuoni	Muer	103° 32′ 10″	34° 34′ 17″	2538

1150	Gannan	Zhuoni	Keerqing	103° 26′ 13″	34° 34′ 48″	2539
1151	Gannan	Zhuoni	Keerqing	103° 19′ 05″	34° 37′ 31″	2614
1152	Gannan	Zhuoni	Keerqing	103° 23′ 47″	34° 35′ 33″	2569
1153	Gannan	Zhuoni	Keerqing	103° 20′ 36″	34° 33′ 45″	2685
1154	Gannan	Zhuoni	Keerqing	103° 20′ 24″	34° 33′ 18″	2663
1155	Longnan	Tanchang	Bali	104° 24′ 20″	34° 18′ 35″	2550
1156	Longnan	Tanchang	Bali	104° 24′ 21″	34° 18′ 26″	2585
1157	Longnan	Tanchang	Bali	104° 24′ 23″	34° 18′ 26″	2583
1158	Longnan	Tanchang	Bali	104° 23′ 04″	34° 17′ 58″	2449
1159	Longnan	Tanchang	Bali	104° 23′ 54″	34° 17′ 22″	2510
1160	Longnan	Tanchang	Muerxiang	104° 20′ 18″	34° 16′ 18″	2360
1161	Longnan	Tanchang	Muerxiang	104° 20′ 26″	34° 16′ 18″	2363
1162	Longnan	Tanchang	Lichuan	104° 18′ 55″	34° 17′ 01″	2407
1163	Longnan	Tanchang	Lichuan	104° 18′ 56″	34° 17′ 01″	2454
1164	Longnan	Tanchang	Lichuan	104° 18′ 56″	34° 17′ 21″	2558
1165	Longnan	Tanchang	Bali	104° 17′ 56″	34° 20′ 01″	2531
1166	Longnan	Tanchang	Bali	104° 18′ 26″	34° 19′ 34″	2483
1167	Longnan	Tanchang	Bali	104° 18′ 23″	34° 19′ 18″	2498
1168	Longnan	Tanchang	Bali	104° 18′ 27″	34° 18′ 14″	2497
1169	Longnan	Tanchang	Bali	104° 20′ 32″	34° 18′ 18″	2339
1170	Longnan	Tanchang	Bali	104° 19′ 42″	34° 19′ 57″	2512
1171	Longnan	Tanchang	Bali	104° 19′ 44″	34° 19′ 49″	2501
1172	Longnan	Tanchang	Bali	104° 22′ 39″	34° 18′ 44″	2433
1173	Longnan	Tanchang	Bali	104° 23′ 00″	34° 18′ 56″	2447
1174	Longnan	Tanchang	Bali	104° 23′ 27″	34° 19′ 00″	2476
1175	Longnan	Tanchang	Pangjiaxiang	104° 12′ 30″	34° 20′ 44″	2666
1176	Longnan	Tanchang	Pangjiaxiang	104° 12′ 31″	34° 20′ 43″	2646
1177	Longnan	Tanchang	Pangjiaxiang	104° 15′ 21″	34° 19′ 17″	2537
1178	Longnan	Tanchang	Pangjiaxiang	104° 15′ 24″	34° 19′ 16″	2539
1179	Longnan	Tanchang	Pangjiaxiang	104° 15′ 31″	34° 18′ 57″	2396
1180	Longnan	Tanchang	Pangjiaxiang	104° 16′ 41″	34° 18′ 50″	2408
1181	Longnan	Tanchang	Pangjiaxiang	104° 16′ 41″	34° 18′ 47″	2407
1182	Longnan	Tanchang	Lichuan	104° 17′ 16″	34° 15′ 20″	2243
1183	Longnan	Tanchang	Lichuan	104° 16′ 53″	34° 15′ 05″	2294
1184	Longnan	Tanchang	Lichuan	104° 16′ 39″	34° 15′ 19″	2345
1185	Longnan	Tanchang	Lichuan	114° 63′ 30″	34° 15′ 20″	1520
1186	Longnan	Tanchang	Lichuan	104° 16′ 30″	34° 15′ 19″	1519
1187	Longnan	Tanchang	Lichuan	104° 20′ 03″	34° 10′ 11″	2320
1188	Longnan	Tanchang	Lichuan	104° 20′ 00″	34° 10′ 12″	2302
1189	Longnan	Tanchang	Lichuan	104° 20′ 45″	34° 12′ 22″	2349
1190	Longnan	Tanchang	Lichuan	104° 20′ 42″	34° 12′ 38″	2468
1191	Longnan	Tanchang	Lichuan	104° 20′ 45″	34° 12′ 45″	2482
1192	Longnan	Tanchang	Lichuan	104° 20′ 47″	34° 12′ 47″	2488
1193	Longnan	Tanchang	Lichuan	104° 20′ 52″	34° 12′ 48″	2482
1194	Longnan	Tanchang	Lichuan	104° 20′ 52″	34° 12′ 47″	2493
1195	Longnan	Tanchang	Lichuan	104° 20′ 41″	34° 12′ 37″	2428
1196	Longnan	Tanchang	Lichuan	104° 19′ 16″	34° 13′ 03″	2245
1197	Longnan	Tanchang	Lichuan	104° 19′ 20″	34° 13′ 04″	2475

1198	Longnan	Tanchang	Lichuan	104° 17′ 29″	34° 13′ 47″	2223
1199	Longnan	Tanchang	Lichuan	104° 17′ 13″	34° 12′ 46″	2474
1200	Longnan	Tanchang	Lichuan	104° 17′ 13″	34° 12′ 47″	2474
1201	Longnan	Tanchang	Hadapu	104° 11′ 51″	34° 15′ 30″	2382
1202	Longnan	Tanchang	Hadapu	104° 11′ 51″	34° 15′ 30″	2384
1203	Longnan	Tanchang	Jiahexiang	104° 24′ 06″	34° 12′ 34″	2395
1204	Longnan	Tanchang	Jiahexiang	104° 25′ 29″	34° 12′ 30″	2627
1205	Longnan	Tanchang	Jiahexiang	104° 25′ 42″	34° 12′ 26″	2691
1206	Longnan	Tanchang	Jiahexiang	104° 25′ 22″	34° 12′ 02″	2771
1207	Longnan	Tanchang	Jiahexiang	104° 25′ 15″	34° 12′ 09″	2769
1208	Longnan	Tanchang	Jiahexiang	104° 25′ 09″	34° 12′ 15″	2699
1209	Longnan	Tanchang	Jiahexiang	104° 25′ 12″	34° 12′ 17″	2713
1210	Longnan	Tanchang	Jiahexiang	104° 25′ 19″	34° 11′ 50″	2728
1211	Longnan	Tanchang	Jiahexiang	104° 25′ 41″	34° 12′ 32″	2712
1212	Longnan	Tanchang	Jiahexiang	104° 23′ 40″	34° 11′ 01″	2515
1213	Longnan	Tanchang	Jiahexiang	104° 23′ 37″	34° 10′ 59″	2511
1214	Longnan	Tanchang	Jiahexiang	104° 23′ 49″	34° 11′ 03″	2520
1215	Longnan	Tanchang	Jiahexiang	104° 24′ 50″	34° 11′ 02″	2777
1216	Longnan	Tanchang	Jiahexiang	104° 24′ 46″	34° 11′ 09″	2729
1217	Longnan	Tanchang	Jiahexiang	104° 21′ 57″	34° 10′ 19″	2385
1218	Longnan	Tanchang	Jiahexiang	104° 21′ 24″	34° 10′ 40″	2507
1219	Longnan	Tanchang	Jiahexiang	104° 22′ 18″	34° 09′ 54″	2207
1220	Longnan	Tanchang	Jiahexiang	104° 21′ 34″	34° 09′ 07″	2392
1221	Longnan	Tanchang	Jiahexiang	104° 21′ 38″	34° 09′ 04″	2390
1222	Longnan	Tanchang	Jiahexiang	104° 20′ 33″	34° 05′ 53″	2383
1223	Longnan	Tanchang	Jiahexiang	104° 17′ 47″	34° 06′ 57″	2330
1224	Longnan	Tanchang	Jiahexiang	104° 17′ 48″	34° 06′ 57″	2330
1225	Longnan	Tanchang	Jiangtaixiang	104° 26′ 09″	34° 06′ 35″	2503
1226	Longnan	Tanchang	Jiangtaixiang	104° 26′ 16″	34° 06′ 35″	2515
1227	Longnan	Tanchang	Jiangtaixiang	104° 26′ 50″	34° 07′ 43″	2532
1228	Longnan	Tanchang	Jiangtaixiang	104° 26′ 48″	34° 07′ 43″	2533
1229	Longnan	Tanchang	Jiangtaixiang	104° 24′ 43″	34° 07′ 32″	2190
1230	Longnan	Tanchang	Jiangtaixiang	104° 24′ 49″	34° 04′ 56″	2267
1231	Longnan	Tanchang	Xinghuaxiang	104° 35′ 29″	34° 02′ 43″	2300
1232	Longnan	Tanchang	Xinghuaxiang	104° 35′ 16″	34° 02′ 44″	2360
1233	Longnan	Tanchang	Xinghuaxiang	104° 37′ 58″	34° 02′ 08″	2359
1234	Longnan	Tanchang	Xinghuaxiang	104° 37′ 58″	34° 08′ 19″	2364
1235	Longnan	Tanchang	Xinghuaxiang	104° 37′ 50″	34° 08′ 18″	2364
1236	Longnan	Tanchang	Xinghuaxiang	104° 37′ 48″	34° 08′ 17″	2365
1237	Longnan	Tanchang	Xinghuaxiang	104° 34′ 30″	34° 06′ 38″	2382
1238	Longnan	Tanchang	Chelaxiang	104° 31′ 38″	34° 06′ 48″	2545
1239	Longnan	Tanchang	Chelaxiang	104° 27′ 44″	34° 08′ 06″	2414
1240	Longnan	Tanchang	Chelaxiang	104° 27′ 45″	34° 08′ 06″	2420
1241	Longnan	Tanchang	Chelaxiang	104° 28′ 39″	34° 06′ 39″	2164
1242	Longnan	Tanchang	Muerxiang	104° 21′ 25″	34° 13′ 47″	2405
1243	Longnan	Tanchang	Muerxiang	104° 21′ 26″	34° 13′ 48″	2415
1244	Longnan	Tanchang	Muerxiang	104° 21′ 25″	34° 13′ 55″	2354
1245	Longnan	Tanchang	Muerxiang	104° 21′ 32″	34° 14′ 03″	2389

1246	Longnan	Tanchang	Muerxiang	104° 21′ 42″	34° 14′ 45″	2364
1247	Longnan	Tanchang	Muerxiang	104° 22′ 43″	34° 16′ 01″	2526
1248	Longnan	Tanchang	Muerxiang	104° 22′ 37″	34° 16′ 01″	2574
1249	Longnan	Tanchang	Muerxiang	104° 21′ 15″	34° 14′ 44″	2433
1250	Longnan	Tanchang	Muerxiang	104° 20′ 55″	34° 14′ 30″	2440
1251	Longnan	Tanchang	Muerxiang	104° 20′ 54″	34° 14′ 02″	2418
1252	Longnan	Tanchang	Hadapu	104° 11′ 39″	34° 17′ 56″	2447
1253	Longnan	Tanchang	Hadapu	104° 11′ 44″	34° 17′ 53″	2441
1254	Longnan	Tanchang	Hadapu	104° 12′ 17″	34° 17′ 50″	2385
1255	Longnan	Tanchang	Hadapu	104° 12′ 19″	34° 18′ 08″	2435
1256	Longnan	Tanchang	Hadapu	104° 12′ 52″	34° 17′ 27″	2384
1257	Longnan	Tanchang	Hadapu	104° 14′ 30″	34° 16′ 46″	2377
1258	Longnan	Tanchang	Hadapu	104° 14′ 32″	34° 16′ 49″	2385
1259	Longnan	Tanchang	Hadapu	104° 14′ 48″	34° 16′ 29″	2320
1260	Longnan	Tanchang	Awuzheng	104° 09′ 37″	34° 16′ 15″	2341
1261	Longnan	Tanchang	Awuzheng	104° 09′ 10″	34° 16′ 12″	2372
1262	Longnan	Tanchang	Awuzheng	104° 10′ 43″	34° 15′ 09″	2315
1263	Longnan	Tanchang	Awuzheng	104° 10′ 31″	34° 15′ 34″	2289
1264	Longnan	Tanchang	Awuzheng	104° 11′ 16″	34° 16′ 22″	2385
1265	Longnan	Tanchang	Awuzheng	104° 10′ 15″	34° 19′ 35″	2533
1266	Longnan	Tanchang	Awuzheng	104° 10′ 04″	34° 19′ 58″	2588
1267	Longnan	Tanchang	Awuzheng	104° 09′ 35″	34° 17′ 55″	2483
1268	Longnan	Tanchang	Awuzheng	104° 09′ 35″	34° 17′ 56″	2507
1269	Longnan	Tanchang	Awuzheng	104° 09′ 01″	34° 17′ 33″	2363
1270	Longnan	Tanchang	Awuzheng	104° 09′ 04″	34° 17′ 34″	2383
1271	Longnan	Tanchang	Awuzheng	104° 08′ 59″	34° 17′ 21″	2368
1272	Longnan	Tanchang	Awuzheng	104° 07′ 53″	34° 16′ 45″	2505
1273	Longnan	Tanchang	Awuzheng	104° 07′ 51″	34° 16′ 59″	2491
1274	Longnan	Tanchang	Awuzheng	104° 09′ 54″	34° 17′ 03″	2340
1275	Longnan	Tanchang	Muerzheng	104° 20′ 58″	34° 15′ 02″	2425
1276	Longnan	Tanchang	Muerzheng	104° 21′ 14″	34° 16′ 04″	2386
1277	Longnan	Tanchang	Muerzheng	104° 21′ 23″	34° 16′ 01″	2436
1278	Longnan	Tanchang	Nanhezheng	104° 09′ 52.60″	34° 03′ 31.44″	2155
1279	Longnan	Tanchang	Nanhezheng	104° 10′ 16.58″	34° 05′ 01.55″	2083
1280	Longnan	Tanchang	Nanhezheng	104° 11′ 06.94″	34° 05′ 54.18″	2083
1281	Longnan	Tanchang	Nanhezheng	104° 11′ 12.78″	34° 05′ 51.85″	2073
1282	Longnan	Lixian	Qiaotou	104° 08′ 05.62″	33° 09′ 65.34″	1900
1283	Longnan	Lixian	Qiaotou	104° 48′ 31.95″	33° 43′ 18.59″	1950
1284	Longnan	Lixian	Shajin	104° 43′ 20.75″	34° 05′ 54.98″	2443
1285	Longnan	Lixian	Shajin	104° 47′ 37.42″	34° 00′ 05.74″	1917
1286	Longnan	Lixian	Shajin	104° 48′ 25.43″	33° 59′ 37.69″	1906
1287	Longnan	Lixian	Shajin	104° 48′ 27.67″	33° 59′ 88.20″	1906
1288	Longnan	Lixian	Shajin	104° 48′ 41.35″	33° 58′ 33.52″	1910
1289	Longnan	Lixian	Shajin	104° 49′ 48.82″	33° 59′ 42.58″	1971
1290	Longnan	Lixian	Shajin	104° 49′ 41.92″	34° 00′ 08.83″	1991
1291	Longnan	Lixian	Shajin	104° 49′ 57.10″	34° 01′ 28.52″	2072
1292	Longnan	Lixian	Qiushan	104° 51′ 42.62″	34° 21′ 31.00″	2080
1293	Longnan	Lixian	Qiushan	104° 51′ 20.54″	34° 21′ 02.11″	2040

1294	Longnan	Lixian	Qiushan	104° 53′ 06.94″	34° 21′ 19.01″	2002
1295	Longnan	Lixian	Qiushan	104° 54′ 45.39″	34° 19′ 29.57″	2011
1296	Longnan	Lixian	Taoping	104° 54′ 21.54″	34° 17′ 18.33″	2240
1297	Longnan	Lixian	Qiaotou	104° 48′ 42.86″	33° 41′ 03.62″	2100
1298	Longnan	Lixian	Caoping	104° 48′ 35.65″	33° 41′ 18.78″	2302
1299	Longnan	Lixian	Caoping	104° 48′ 00.50″	33° 40′ 38.63″	2320
1300	Longnan	Lixian	Caoping	104° 47′ 31.39″	33° 40′ 25.10″	2570
1301	Longnan	Lixian	Caoping	104° 46′ 48.70″	33° 40′ 12.75″	2567
1302	Longnan	Lixian	Caoping	104° 46′ 26.38″	33° 40′ 29.12″	2574
1303	Longnan	Lixian	Caoping	104° 45′ 44.84″	33° 39′ 35.86″	2578
1304	Longnan	Lixian	Caoping	104° 50′ 51.00″	33° 39′ 30.00″	2526
1305	Longnan	Lixian	Caoping	104° 51′ 31.00″	33° 40′ 12.00″	2487
1306	Longnan	Lixian	Caoping	104° 51′ 49.00″	33° 40′ 48.00″	2480
1307	Longnan	Lixian	Caoping	104° 48′ 59.79″	33° 47′ 13.73″	2276
1308	Longnan	Lixian	Caoping	104° 48′ 45.63″	33° 39′ 32.38″	2557
1309	Longnan	Lixian	Caoping	104° 48′ 41.25″	33° 39′ 31.67″	2541
1310	Longnan	Lixian	Caoping	104° 48′ 46.35″	33° 41′ 00.91″	2276
1311	Longnan	Wudu	Chiba	104° 42′ 50.66″	33° 38′ 10.55″	2549
1312	Longnan	Wudu	Chiba	104° 43′ 57.62″	33° 38′ 11.08″	2697
1313	Longnan	Wudu	Chiba	104° 47′ 07.92″	33° 38′ 31.70″	2465
1314	Longnan	Wudu	Chiba	104° 48′ 46.33″	33° 38′ 28.23″	2276
1315	Longnan	Wudu	Chiba	104° 45′ 20.36″	33° 38′ 09.89″	2573
1316	Longnan	Wudu	Chiba	104° 45′ 37.57″	33° 37′ 49.09″	2549
1317	Longnan	Wudu	Chiba	104° 45′ 22.57″	33° 37′ 17.01″	2579
1318	Longnan	Wudu	Chiba	104° 45′ 16.41″	33° 37′ 17.26″	2590
1319	Longnan	Wudu	Chiba	104° 46′ 21.59″	33° 37′ 42.37″	2523
1320	Longnan	Wudu	Chiba	104° 47′ 15.47″	33° 37′ 24.10″	2430
1321	Longnan	Wudu	Chiba	104° 47′ 41.86″	33° 37′ 40.94″	2433
1322	Longnan	Wudu	Chiba	104° 47′ 38.46″	33° 37′ 34.91″	2435
1323	Longnan	Wudu	Chiba	104° 47′ 47.93″	33° 38′ 14.97″	2462
1324	Longnan	Wudu	Chiba	104° 48′ 42.20″	33° 38′ 12.49″	2474
1325	Longnan	Wudu	Maying	104° 49′ 04.18″	33° 37′ 03.02″	2447
1326	Longnan	Wudu	Maying	104° 49′ 06.49″	33° 37′ 03.31″	2482
1327	Longnan	Wudu	Maying	104° 49′ 03.54″	03° 36′ 30.07″	2415
1328	Longnan	Wudu	Maying	104° 49′ 02.53″	33° 36′ 52.51″	2490
1329	Longnan	Wudu	Maying	104° 50′ 14.08″	33° 36′ 22.78″	2508
1330	Longnan	Wudu	Maying	104° 50′ 40.42″	33° 35′ 55.64″	2446
1331	Longnan	Wudu	Maying	104° 50′ 55.40″	33° 35′ 01.04″	2380
1332	Longnan	Wudu	Maying	104° 51′ 08.50″	33° 34′ 31.32″	2355
1333	Longnan	Wudu	Maying	104° 50′ 23.20″	33° 33′ 51.78″	2208
1334	Longnan	Wudu	Maying	104° 50′ 24.77″	33° 33′ 44.88″	2190
1335	Longnan	Wudu	Maying	104° 50′ 20.17″	33° 33′ 41.60″	2082
1336	Longnan	Wudu	Maying	104° 50′ 19.55″	33° 33′ 32.53″	2035
1337	Longnan	Wudu	马街	104° 53′ 21.42″	33° 33′ 42.23″	2190
1338	Longnan	Wudu	马街	104° 53′ 17.85″	33° 33′ 49.70″	2082
1339	Longnan	Wudu	马街	104° 54′ 57.61″	33° 32′ 36.58″	2035
1340	Longnan	Wudu	马街	104° 55′ 01.84″	33° 32′ 45.81″	2007
1341	Longnan	Wudu	Maying	104° 54′ 06.00″	33° 33′ 43.00″	2190

1342	Longnan	Wudu	Maying	104° 53′ 17.85″	33° 33′ 49.70″	2082
1343	Longnan	Wudu	Maying	104° 53′ 39.00″	33° 35′ 16.00″	2378
1344	Longnan	Wudu	Maying	104° 53′ 41.16″	33° 33′ 40.27″	2343
1345	Longnan	Wudu	Maying	104° 53′ 39.48″	33° 35′ 21.05″	2195
1346	Longnan	Wudu	Maying	104° 52′ 17.92″	33° 34′ 33.45″	2378
1347	Longnan	Wudu	Maying	104° 52′ 03.80″	33° 34′ 47.25″	2343
1348	Longnan	Wudu	Maying	104° 52′ 17.32″	33° 35′ 31.21″	2359
1349	Longnan	Wudu	Maying	104° 50′ 44.89″	33° 35′ 54.59″	2370
1350	Longnan	Wudu	Maying	104° 51′ 58.26″	33° 37′ 05.08″	2377
1351	Longnan	Wudu	Maying	104° 52′ 57.52″	33° 37′ 55.30″	2372
1352	Longnan	Wudu	Maying	104° 53′ 01.69″	33° 38′ 12.47″	2322
1353	Longnan	Wudu	Maying	104° 54′ 12.30″	33° 39′ 45.11″	2294
1354	Longnan	Wudu	Maying	104° 54′ 12.30″	33° 39′ 45.11″	2294
1355	Longnan	Wudu	Maying	104° 54′ 57.61″	33° 32′ 36.58″	2045
1356	Longnan	Wudu	Maying	104° 50′ 03.31″	33° 33′ 56.03″	2051
1357	Longnan	Wudu	Puchi	104° 49′ 22.18″	33° 32′ 24.33″	2056
1358	Longnan	Wudu	Puchi	104° 49′ 36.17″	33° 32′ 12.16″	2011
1359	Longnan	Wudu	Puchi	104° 45′ 22.91″	33° 33′ 43.59″	2014
1360	Longnan	Wudu	Puchi	104° 44′ 53.62″	33° 34′ 27.11″	2170
1361	Longnan	Wudu	Puchi	104° 45′ 22.91″	33° 33′ 43.59″	2168
1362	Longnan	Wenxian	Buziba	104° 39′ 39.00″	33° 08′ 16.00″	1865
1363	Longnan	Wenxian	Buziba	104° 37′ 15.00″	33° 07′ 24.00″	1990
1364	Longnan	Wenxian	Buziba	104° 41′ 10.00″	33° 09′ 24.00″	2020
1365	Longnan	Wenxian	Buziba	104° 41′ 11.00″	33° 09′ 23.00″	2014
1366	Longnan	Wenxian	Buziba	104° 40′ 10.00″	33° 09′ 15.00″	1836
1367	Longnan	Wenxian	Qiaotou	104° 42′ 47.00″	33° 03′ 56.00″	1736
1368	Linxia	Kangle	Wanghu	103° 68′ 56″	35° 15′ 05″	2246
1369	Linxia	Kangle	Wanghu	103° 68′ 33″	35° 15′ 95″	2248
1370	Linxia	Kangle	Wanghu	103° 68′ 30″	35° 16′ 00″	2249
1371	Linxia	Kangle	Wanghu	103° 69′ 40″	35° 14′ 46″	2145
1372	Linxia	Kangle	Wanghu	103° 69′ 95″	35° 16′ 09″	2220
1373	Linxia	Kangle	Wanghu	103° 69′ 12″	35° 16′ 98″	2186
1374	Linxia	Kangle	Wanghu	103° 69′ 42″	35° 17′ 19″	2193
1375	Linxia	Kangle	Wanghu	103° 66′ 24″	35° 18′ 03″	2274
1376	Linxia	Kangle	Wanghu	103° 66′ 20″	35° 19′ 36″	2311
1377	Linxia	Kangle	Wanghu	103° 69′ 27″	35° 19′ 55″	2318
1378	Linxia	Kangle	Wanghu	103° 69′ 23″	35° 19′ 42″	2306
1379	Linxia	Kangle	Wanghu	103° 70′ 18″	35° 17′ 33″	2257
1380	Linxia	Kangle	Wanghu	103° 49′ 01″	35° 17′ 21″	2239
1381	Linxia	Kangle	Jinggu	103° 65′ 83″	35° 12′ 16″	2329
1382	Linxia	Kangle	Jinggu	103° 65′ 88″	35° 12′ 18″	2333
1383	Linxia	Kangle	Jinggu	103° 65′ 28″	35° 12′ 06″	2368
1384	Linxia	Kangle	Jinggu	103° 65′ 15″	35° 12′ 04″	2388
1385	Linxia	Kangle	Jinggu	103° 65′ 40″	34° 12′ 19″	2375
1386	Linxia	Kangle	Jinggu	103° 65′ 63″	34° 12′ 10″	2324
1387	Linxia	Kangle	Jinggu	103° 65′ 23″	35° 15′ 44″	2263
1388	Linxia	Kangle	Jinggu	103° 63′ 97″	35° 15′ 84″	2296
1389	Linxia	Kangle	Jinggu	103° 67′ 95″	35° 11′ 77″	2237

1390	Linxia	Kangle	Jinggu	103° 68′ 06″	35° 11′ 71″	2227
1391	Linxia	Kangle	Jinggu	103° 66′ 50″	35° 11′ 12″	2240
1392	Linxia	Kangle	Jinggu	103° 66′ 37″	35° 11′ 06″	2255
1393	Linxia	Kangle	Jinggu	103° 67′ 31″	35° 05′ 87″	2204
1394	Linxia	Kangle	Jinggu	103° 67′ 84″	35° 07′ 94″	2247
1395	Linxia	Kangle	Jinggu	103° 70′ 58″	35° 07′ 04″	2252
1396	Linxia	Kangle	Caotan	103° 64′ 58″	35° 20′ 57″	2339
1397	Linxia	Kangle	Caotan	103° 64′ 51″	35° 20′ 66″	2365
1398	Linxia	Kangle	Caotan	103° 64′ 08″	35° 20′ 22″	2388
1399	Linxia	Kangle	Caotan	103° 63′ 82″	35° 20′ 07″	2412
1400	Linxia	Kangle	Caotan	103° 63′ 07″	35° 21′ 63″	2413
1401	Linxia	Kangle	Caotan	103° 62′ 28″	35° 21′ 41″	2429
1402	Linxia	Kangle	Caotan	103° 61′ 39″	35° 21′ 57″	2455
1403	Linxia	Kangle	Shangwan	103° 59′ 02″	35° 24′ 81″	2287
1404	Linxia	Kangle	Caotan	103° 59′ 10″	35° 24′ 64″	2292
1405	Linxia	Kangle	Caotan	103° 60′ 22″	35° 25′ 74″	2251
1406	Linxia	Kangle	Basong	103° 48′ 99″	35° 33′ 27″	2489
1407	Linxia	Kangle	Basong	103° 49′ 31″	35° 33′ 85″	2495
1408	Linxia	Kangle	Basong	104° 48′ 05″	35° 33′ 71″	2400
1409	Linxia	Kangle	Basong	103° 46′ 93″	35° 32′ 32″	2452
1410	Linxia	Kangle	Basong	103° 48′ 37″	35° 30′ 73″	2252
1411	Linxia	Kangle	Basong	103° 47′ 27″	35° 29′ 34″	2282
1412	Linxia	Kangle	Basong	103° 47′ 42″	35° 29′ 83″	2280
1413	Linxia	Kangle	Basong	103° 49′ 14″	35° 30′ 56″	2250
1414	Linxia	Kangle	Basong	103° 48′ 79″	35° 29′ 14″	2287
1415	Linxia	Kangle	Basong	103° 46′ 25″	35° 27′ 76″	2324
1416	Linxia	Kangle	Minglu	103° 32′ 09″	35° 16′ 50″	2259
1417	Linxia	Kangle	Minglu	103° 32′ 10″	35° 16′ 56″	2277
1418	Linxia	Kangle	Minglu	103° 31′ 58″	35° 16′ 46″	2292
1419	Linxia	Kangle	Suji	103° 31′ 04″	35° 20′ 02″	2331
1420	Linxia	Kangle	Baiwang	10° 33′ 42″	35° 21′ 02″	2354
1421	Linxia	Kangle	Baiwang	103° 31′ 42″	35° 21′ 10″	2339
1422	Linxia	Kangle	Baiwang	103° 33′ 12″	35° 22′ 51″	2236
1423	Linxia	Kangle	Lianlu	103° 42′ 43″	35° 03′ 06″	2285
1424	Linxia	Kangle	Lianlu	103° 42′ 38″	35° 03′ 31″	2308
1425	Linxia	Kangle	Lianlu	103° 02′ 54″	35° 02′ 54″	2301
1426	Linxia	Kangle	Lianlu	103° 45′ 01″	34° 59′ 59″	2039
1427	Linxia	Kangle	Lianlu	103° 44′ 46″	34° 59′ 38″	2027
1428	Linxia	Kangle	Lianlu	103° 46′ 19″	35° 01′ 02″	1993
1429	Linxia	Lingxia	Zhangzigou	102° 97′ 14″	35° 53′ 87″	2511
1430	Linxia	Lingxia	Zhangzigou	102° 94′ 97″	35° 53′ 69″	2540
1431	Linxia	Lingxia	monigou	102° 89′ 82″	35° 46′ 06″	2438
1432	Linxia	Lingxia	monigou	102° 90′ 17″	35° 46′ 66″	2402
1433	Linxia	Lingxia	monigou	102° 91′ 37″	35° 47′ 50″	2371
1434	Linxia	Lingxia	Yanji	103° 06′ 66″	35° 38′ 67″	2271
1435	Linxia	Lingxia	Yanji	103° 06′ 78″	35° 38′ 63″	2266
1436	Linxia	Meijishan	Zhaizigou	102° 86′ 38″	35° 69′ 21″	2397
1437	Linxia	Meijishan	Zhaizigou	102° 86′ 19″	35° 69′ 19″	2395

1438	Linxia	Meijishan	Zhaizigou	102° 85′ 52″	35° 68′ 92″	2417
1439	Linxia	Meijishan	Zhaizigou	102° 86′ 31″	35° 69′ 07″	2380
1440	Linxia	Meijishan	Zhaizigou	102° 84′ 94″	35° 67′ 97″	2466
1441	Linxia	Meijishan	Chuimatan	102° 83′ 47″	35° 70′ 74″	2434
1442	Linxia	Meijishan	Liugou	102° 86′ 89″	35° 73′ 61″	2481
1443	Linxia	Meijishan	Xiaoguan	102° 89′ 95″	35° 57′ 88″	2314
1444	Wuwei	Tianzhu	Songshan	102° 17′ 25″	37° 00′ 29″	2633
1445	Wuwei	Tianzhu	Dachaigou	102° 52′ 06″	37° 07′ 10″	2865
1446	Wuwei	Tianzhu	Duoshi	102° 56′ 18″	37° 15′ 85″	2617
1447	Wuwei	Tianzhu	Duoshi	102° 57′ 08″	37° 18′ 56″	2552
1448	Wuwei	Tianzhu	Duoshi	103° 56′ 44″	37° 18′ 16″	2694
1449	Wuwei	Tianzhu	Duoshi	103° 58′ 66″	37° 18′ 20″	2544
1450	Wuwei	Tianzhu	Duoshi	103° 00′ 46″	37° 19′ 95″	2564
1451	Wuwei	Tianzhu	Duoshi	103° 59′ 26″	37° 18′ 99″	2615
1452	Wuwei	Tianzhu	Duoshi	103° 00′ 60″	37° 18′ 44″	2613
1453	Wuwei	Tianzhu	Duoshi	103° 02′ 13″	37° 18′ 07″	2752
1454	Wuwei	Tianzhu	Duoshi	103° 03′ 43″	37° 28′ 56″	2690
1455	Wuwei	Tianzhu	Duoshi	103° 02′ 19″	37° 18′ 12″	2762
1456	Wuwei	Tianzhu	Duoshi	103° 03′ 03″	37° 19′ 09″	2619
1457	Wuwei	Tianzhu	Duoshi	103° 04′ 00″	37° 18′ 17″	2647
1458	Wuwei	Tianzhu	Duoshi	103° 04′ 93″	37° 18′ 45″	2666
1459	Wuwei	Tianzhu	Xidatan	103° 06′ 10″	37° 17′ 17″	2789
1460	Wuwei	Tianzhu	Xidatan	103° 07′ 44″	37° 17′ 22″	2823
1461	Wuwei	Tianzhu	Xidatan	103° 09′ 88″	37° 20′ 99″	2798
1462	Wuwei	Tianzhu	Xidatan	103° 09′ 00″	37° 21′ 18″	2731
1463	Wuwei	Tianzhu	Xidatan	103° 10′ 84″	37° 21′ 72″	2715
1464	Wuwei	Tianzhu	Anyuan	102° 49′ 06″	37° 22′ 10″	2608
1465	Wuwei	Tianzhu	Anyuan	102° 47′ 57″	37° 21′ 57″	2718
1466	Wuwei	Tianzhu	Anyuan	102° 49′ 11″	37° 21′ 29″	2653
1467	Wuwei	Tianzhu	Anyuan	102° 48′ 10″	37° 20′ 46″	2820
1468	Wuwei	Tianzhu	Anyuan	102° 51′ 17″	37° 16′ 19″	2788
1469	Wuwei	Tianzhu	Anyuan	102° 51′ 49″	37° 16′ 06″	2618
1470	Wuwei	Tianzhu	Anyuan	102° 51′ 33″	37° 15′ 54″	2606
1471	Wuwei	Tianzhu	Anyuan	102° 50′ 28″	37° 15′ 18″	2674
1472	Wuwei	Tianzhu	Anyuan	102° 50′ 40″	37° 13′ 59″	2762
1473	Wuwei	Tianzhu	Anyuan	102° 50′ 41″	37° 13′ 46″	2784
1474	Wuwei	Tianzhu	Anyuan	102° 50′ 47″	37° 14′ 52″	2698
1475	Wuwei	Tianzhu	Anyuan	102° 50′ 20″	37° 14′ 27″	2713
1476	Wuwei	Tianzhu	Anyuan	102° 51′ 47″	37° 14′ 45″	2769
1477	Wuwei	Tianzhu	Anyuan	102° 51′ 85″	37° 15′ 01″	2706
1478	Wuwei	Tianzhu	Tanshanling	102° 40′ 53″	36° 56′ 28″	2646
1479	Wuwei	Tianzhu	Saishisi	102° 50′ 21″	36° 51′ 07″	2730
1480	Wuwei	Tianzhu	Saishisi	102° 43′ 32″	36° 47′ 43″	2203
1481	Wuwei	Tianzhu	Tiantang	102° 31′ 02″	36° 57′ 34″	2354
1482	Wuwei	Tianzhu	Tiantang	102° 33′ 51″	36° 57′ 10″	2515
1483	Wuwei	Tianzhu	Tiantang	102° 33′ 44″	36° 59′ 00″	2528
1484	Wuwei	Tianzhu	Haxizheng	102° 41′ 67″	37° 24′ 43″	2506
1485	Wuwei	Tianzhu	Haxizheng	102° 51′ 38″	37° 24′ 74″	2534

1486	Wuwei	Tianzhu	Haxizheng	102° 40′ 72″	37° 22′ 49″	2708
1487	Wuwei	Tianzhu	Haxizheng	102° 39′ 62″	37° 22′ 60″	2717
1488	Wuwei	Tianzhu	Haxizheng	102° 38′ 68″	37° 23′ 55″	2656
1489	Wuwei	Tianzhu	Haxizheng	102° 38′ 90″	37° 24′ 60″	2574
1490	Wuwei	Tianzhu	Haxizheng	102° 37′ 87″	37° 25′ 87″	2488
1491	Wuwei	Tianzhu	Haxizheng	102° 37′ 50″	37° 25′ 31″	2487
1492	Wuwei	Tianzhu	Haxizheng	102° 36′ 19″	37° 25′ 48″	2463
1493	Wuwei	Tianzhu	Haxizheng	102° 37′ 65″	37° 23′ 23″	2515
1494	Wuwei	Tianzhu	Dongdatan	103° 22′ 11″	37° 12′ 28″	2778
1495	Wuwei	Tianzhu	Dongdatan	103° 20′ 29″	37° 14′ 09″	2672
1496	Wuwei	Tianzhu	Dongdatan	103° 17′ 46″	37° 14′ 46″	2675
1497	Wuwei	Tianzhu	Huazangsizheng	103° 08′ 07″	37° 01′ 20″	2499
1498	Wuwei	Tianzhu	Huazangsizheng	103° 04′ 17″	37° 02′ 40″	2530
1499	Wuwei	Tianzhu	Huazangsizheng	103° 04′ 22″	37° 02′ 42″	2539
1500	Wuwei	Tianzhu	Huazangsizheng	103° 04′ 55″	37° 01′ 14″	2514
1501	Wuwei	Tianzhu	Xidatan	103° 11′ 30″	37° 19′ 49″	2205
1502	Wuwei	Tianzhu	Xidatan	103° 09′ 79″	37° 21′ 49″	2297
1503	Wuwei	Tianzhu	Haxizheng	102° 42′ 77″	37° 24′ 16″	2444
1504	Wuwei	Tianzhu	Haxizheng	102° 38′ 61″	37° 23′ 53″	2576
1505	Wuwei	Tianzhu	Haxizheng	102° 35′ 76″	37° 26′ 41″	2424
1506	Wuwei	Tianzhu	Haxizheng	102° 35′ 74″	37° 25′ 51″	2480
1507	Wuwei	Tianzhu	Haxizheng	102° 31′ 18″	37° 25′ 72″	2455
1508	Wuwei	Tianzhu	Haxizheng	102° 00′ 00″	37° 26′ 96″	2395
1509	Wuwei	Tianzhu	Dahonggou	102° 35′ 46″	37° 27′ 83″	2380
1510	Wuwei	Tianzhu	Dahonggou	102° 33′ 83″	37° 29′ 79″	2528
1511	Wuwei	Tianzhu	Dahonggou	102° 33′ 44″	37° 29′ 84″	2607
1512	Wuwei	Tianzhu	Haxizheng	102° 42′ 38″	37° 24′ 19″	2486
1513	Wuwei	Gulang	Huangpingchuan	103° 09′ 52″	37° 22′ 23″	2697
1514	Wuwei	Gulang	Huangpingchuan	103° 04′ 08″	37° 21′ 82″	2485
1515	Wuwei	Gulang	Huangpingchuan	103° 01′ 36″	37° 21′ 93″	2485
1516	Wuwei	Gulang	Gufengzheng	102° 50′ 85″	37° 25′ 57″	2246
1517	Wuwei	Gulang	Gufengzheng	102° 50′ 46″	37° 25′ 43″	2256
1518	Wuwei	Gulang	Gufengzheng	102° 50′ 11″	37° 25′ 82″	2258
1519	Zhangye	Mingle	Nanfengzheng	100° 53′ 28″	38° 16′ 53″	2667
1520	Zhangye	Mingle	Nanfengzheng	100° 54′ 12″	38° 16′ 54″	2659
1521	Zhangye	Mingle	Nanfengzheng	100° 56′ 18″	38° 17′ 13″	2669
1522	Zhangye	Mingle	Nanfengzheng	100° 57′ 38″	38° 17′ 05″	2661
1523	Zhangye	Mingle	Nanfengzheng	100° 58′ 35″	38° 17′ 12″	2638
1524	Zhangye	Mingle	Nanfengzheng	100° 00′ 26″	38° 15′ 59″	2689
1525	Zhangye	Mingle	Nanfengzheng	100° 00′ 34″	38° 15′ 58″	2690
1526	Zhangye	Mingle	Nanfengzheng	100° 55′ 27″	38° 16′ 54″	2662
1527	Zhangye	Mingle	Nanfengzheng	100° 24′ 14″	38° 17′ 25″	2618
1528	Zhangye	Sunan	Matixiang	100° 43′ 43″	38° 23′ 43″	2558
1529	Zhangye	Sunan	Matixiang	100° 42′ 43″	38° 23′ 03″	2666
1530	Zhangye	Sunan	Matixiang	100° 48′ 09″	38° 20′ 06″	2629
