# Supplementary figures and images for: Quality suitability regionalization analysis of Angelica sinensis in Gansu, China
Source: PLoS One. 2020 Dec 14;15(12):e0243750. doi: 10.1371/journal.pone.0243750 (PMC7735642; doi:10.1371/journal.pone.0243750)

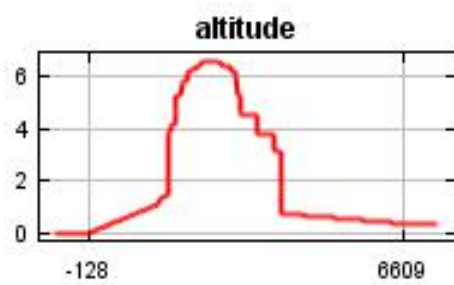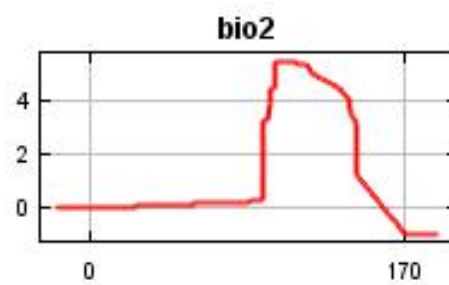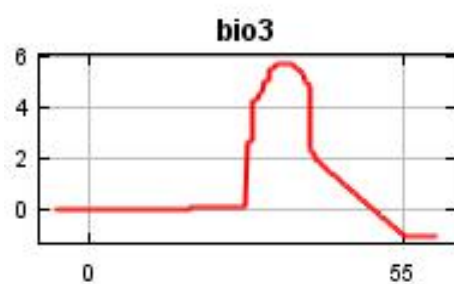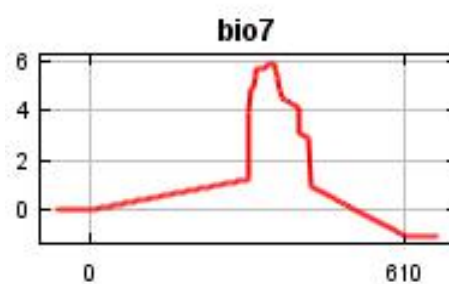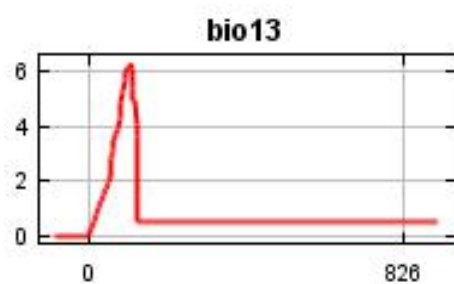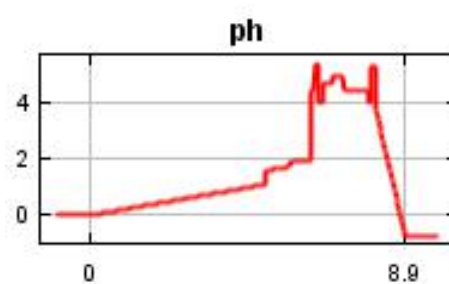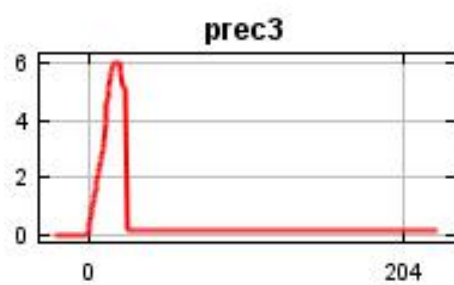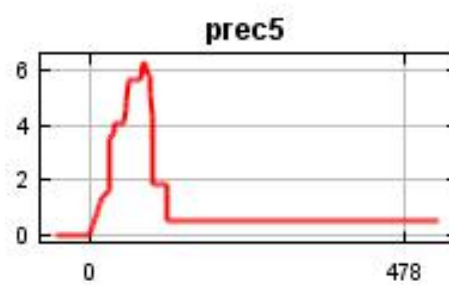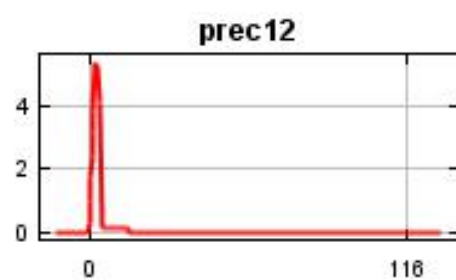

Supplement: S1 File — (PDF) [file pone.0243750.s002.pdf]

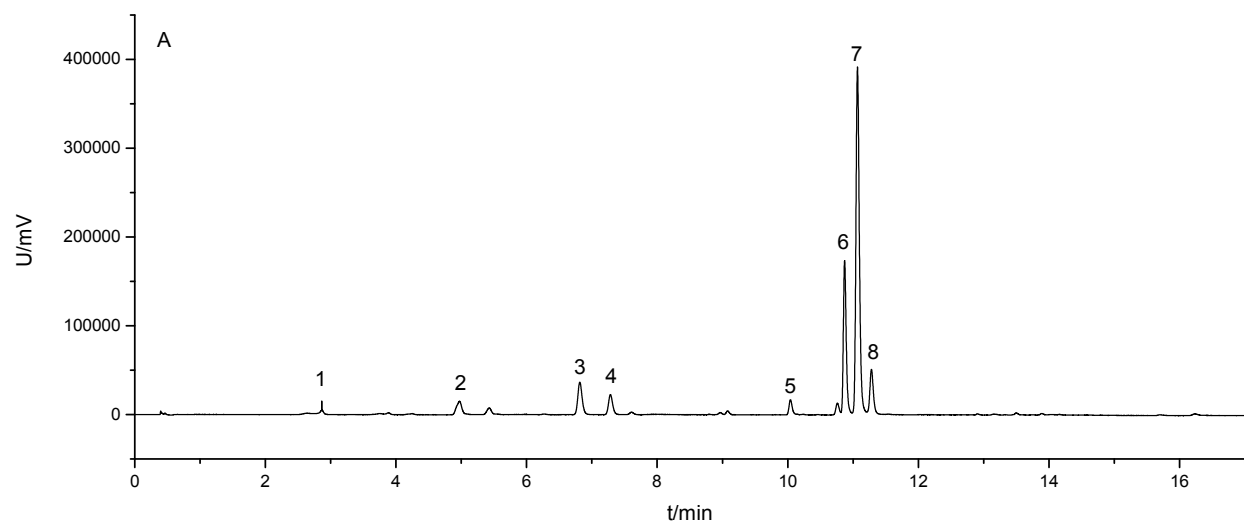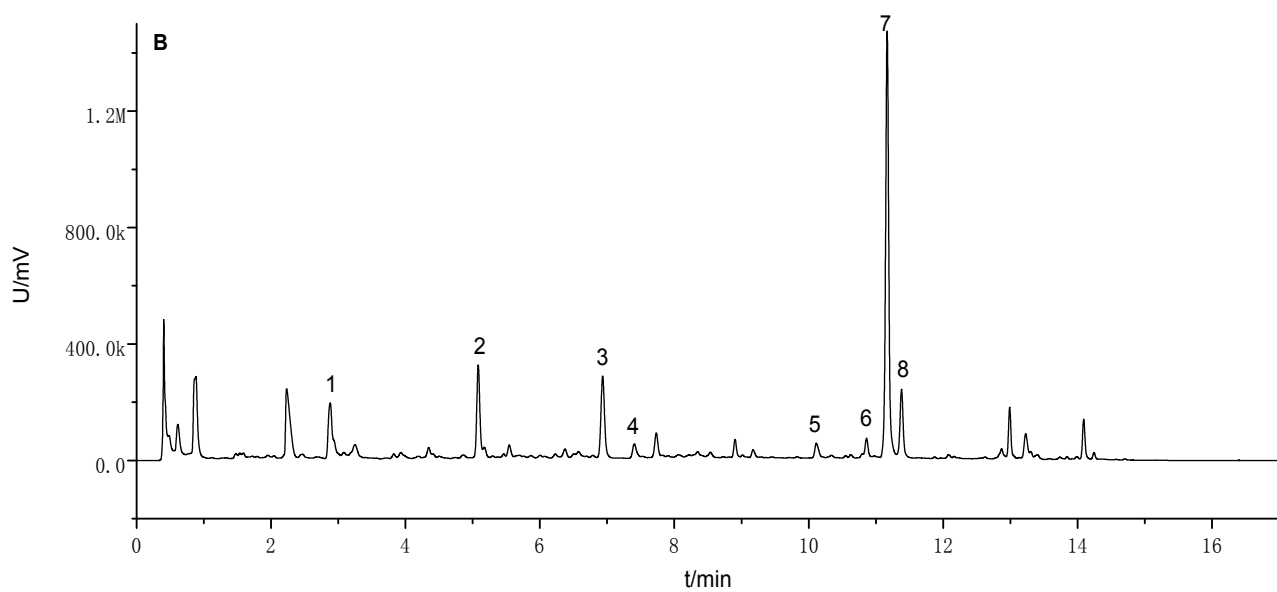

Supplement: S2 File — Note: 1. Chlorogenic acid, 2. Ferulic acid, 3. Senkyunolide I, 4. Senkyunolide H, 5. Senkyunolide A, 6. Coniferyl ferulate, 7. Ligustilide, 8. Butenyl phthalide. (PDF) [file pone.0243750.s003.pdf]
