# Supplementary material for: Quality suitability regionalization analysis of Angelica sinensis in Gansu, China
Source: PLoS One. 2020 Dec 14;15(12):e0243750. doi: 10.1371/journal.pone.0243750 (PMC7735642; doi:10.1371/journal.pone.0243750)
Supplement: Attachment — Submitted filename: Revise Letter.doc [file pone.0243750.s004.doc]

**PONE-D-20-27049 Response to Editors**

18-Dec-2019

Dear Editors:

Thank you for your comments concerning our manuscript entitled “**Association Between Hepatitis B Virus Infection and Risk of Osteoporosis:A Systematic Review and Meta-analysis**”

(ID: **PONE-D-20-27049**). Those comments are all valuable and very helpful for revising and improving our paper, as well as the important guiding significance to our researches. We have studied comments carefully and have made correction which we hope meet with approval. Revised portion are marked in red and blue in the paper.

I have revised the manuscript in a comprehensive way according to your requirements and uploaded relevant documents.

Thank you very much for your kind recommendation, but our research group has a cooperation agreement with MogoEdit, they may revise the language problems of our paper for free, and provide Certification.

We appreciate for Editors warm work earnestly, and hope that the correction will meet with approval.

Once again, thank you very much for your comments and suggestions.

Sincerely,

Xiaoqiong Xu
