# Supplementary material for: Quality suitability regionalization analysis of Angelica sinensis in Gansu, China
Source: PLoS One. 2020 Dec 14;15(12):e0243750. doi: 10.1371/journal.pone.0243750 (PMC7735642; doi:10.1371/journal.pone.0243750)
Supplement: Attachment — Submitted filename: Review comments - Quality suitability regionalization analysis of Angelica sinensis in Gansu.docx [file pone.0243750.s005.docx]

**Review Comments - PONE-D-20-27049**

**Quality suitability regionalization analysis of Angelica sinensis in Gansu, China**

Abstract is in order.

Introduction

In lines 57 – 59, authors should explain how expansion of cultivation of A. Sinensis has deteriorated its yield, since it is expected that when something is expanded, it should be increased in quantity. Otherwise, since focus on this research is on ‘quality’, the yield aspect can be omitted.

Materials and Methods

Lines 93 – 94: What is the difference between meteorological data and comprehensive meteorological data used as ecological factors. I think authors could just use one of these and explain further in the methods the kind of meteorological data used in the study.

Results

Presentation of results in lines 214 – 246 is cumbersome and unclear. Authors should simply present the relationships and their significance coherently, which can be presented in a tabular form to show their predictability.

Discussion

Lines 260 – 295: Authors should back their findings with literature to consolidate generated knowledge from this research. Current discussion is more of a commentary on the results other than backing the results with scientific facts on why such findings were obtained and their implications.

Conclusion is in order.
